# Synthesis, Binding and Fluorescence Studies of Bis-2-amidopyrrole Receptors for Bis-carboxylate Anions

**DOI:** 10.3390/s90301534

**Published:** 2009-03-04

**Authors:** Belén Jiménez, Emilio Calle, Cruz Caballero

**Affiliations:** 1 Organic Chemistry Department, Plaza de los Caidos, 1-5, University of Salamanca, E-37008 Salamanca, Spain; E-Mail: belenjj@yahoo.es; 2 Physical Chemistry Department. Plaza de los Caidos 1-5, University of Salamanca, E-37008 Salamanca, Spain; E-Mail: ecalle@usal.es

**Keywords:** Molecular recognition, bis-2-amidopyrrole receptors, bis-carboxylate anions, fluorescent sensor, hydrogen bonding

## Abstract

The ditopic neutral receptors **1** and **2** were synthesized for the recognition and sensing of bis-carboxylates. They are based on pyrrole and amide groups as hydrogen-bond donors to bind the oxoanions of the guests. Among the obtained results, the selectivity for glutarate over succinate is particularly interesting. Compound **1** behaves as a PET fluorescence sensor for glutarate.

## 1. Introduction

The selective recognition and sensing of anions by synthetic host molecules has become an important area of interest and activity in molecular recognition [1]. Among them, owing to their chemical and biological relevance [2,3] bis-carboxylate anions are attractive targets.

Among a considerable variety of receptors reported in the literature for the binding of carboxylate anions [4], the NH-based hydrogen bonding of heterocyclic compounds has often been employed [5]. Here we report the synthesis and binding properties of new neutral ditopic receptors based on 2-amido-pyrrole moieties as H-bond donors, searching for the selective recognition of bis-carboxylate anions [^−^OOC-(CH_2_)_n_-COO^−^] (n = 2 to 6) on the basis of chain length [6].

**Figure 1.**
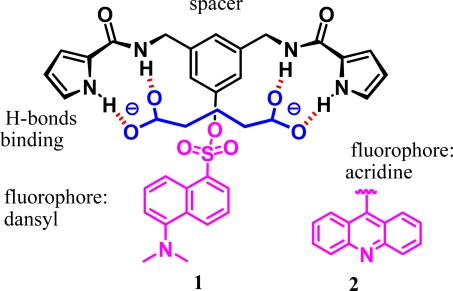
Proposed structure for the complexes of **1** and **2** with glutarate.

Additionally, fluorescent sensors based on the anion-induced changes in fluorescence appear to be particularly attractive due to the simplicity and high detection limit of this tecnique [7]. This accounts for why we adapted our design to develop sensors by introducing a signaling subunit that could detect these guests within the framework of the receptors [8,9]. We describe the symmetrical new derivatives bis-2-pyrroleamide-dansyl **1** and bis-2-pyrroloamide-acridine **2** (Figure 1) as fluorogenic chemosensors, whose photoactive dansyl and acridine groups were connected to the framework of the receptors to supply fluorophore-spacer-receptor systems [10]. The establishment of a host-guest associate affects the electronic properties of the system resulting in optical changes, observables by fluorescence tecniques.

As shown in Figure 1, the binding part of the receptors is composed of two 2-pyrrolecarboxamide groups attached to a *m*-xylylenediamine moiety [11], which acts as a spacer but also as an anchor for introducing the fluorescent dansyl or acridine groups. This spacer could have the appropriate length and relative flexibility for our purposes. The two NH-pyrrole and NH-amide protons stablish hydrogen bond interactions with the negatively charged oxygens of each carboxylate, producing a complex through four H-bonds [12]. ^1^H-NMR titration was used to study the complexation of **1** and **2** with the bis-tetrabutylammonium salts of bis-carboxylates and, in some case, their fluorescence activity was measured.

## 2. Results and Discussion

The synthesis of **1** and **2** is summarized in Scheme 1. 5-Hydroxydimethylisophthalate was protected as the TBDMS derivative **3**, which was reduced to diol **4** and then treated with the iminophosphorane derived from azide under the Staudinger reaction conditions; however, this process failed to produce diamine **5**. On the other hand, the treatment with phthalimide and DIAD/PPh_3_ in THF, using a Mitsunobu protocol [13,14], followed by cleavage of the diphthalimide derivative with hydrazine hydrate in ethanol [15] afforded compound **5**. Additionally, pyrrole was reacted with trichloroacetyl chloride to give the 2-trichloroacylated pyrrole **6**, which was readily coupled with the amine groups of **5** to afford the corresponding diamide **7**. Incorporation of the dansyl or acridine fluorophores was carried out in the final step of the synthesis after deprotection of the phenol, affording the fluorogenic receptors **1** and **2** in 76 and 92 % yield, respectively.

The binding properties [16] of the receptors for the bis-carboxylate anions succinate, glutarate, adipate, pimelate and suberate as bis-TBA salts were investigated by ^1^H-NMR spectroscopy. Titration experiments led to characteristic changes in the ^1^H-NMR spectra that were consistent with the formation of associates.

The results obtained with this procedure provided a satisfactory fit of the titration data to the 1:1 host-guest complex model, the calculated association constants being in the 10^3^ M^−1^ range. This allowed us to confirm the formation of the complexes through four hydrogen bonds (Figure 1).

These studies were carried out in CDCl_3_/DMSO-*d*_6_ (4%) to ensure that the receptors would be completely dissolved during the titration experiments. Gradual addition of small amounts of a guest solution to 1.0 mM solutions of receptors **1** or **2** led to simultaneous downfield shifts in the signals of all NH protons, showing that they are involved in hydrogen bonds, and they always displayed the greatest shifts in all cases studied. Job plot analysis [17] confirmed the stoichiometry of the complexation, a maximum being observed for the complexation of **1** and **2** with all the guests studied at a 0.5 molar fraction, which indicates 1:1 complex formation.

**Figure 2.**
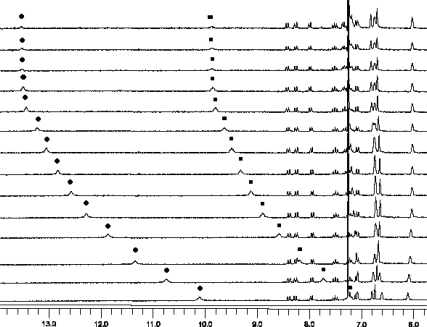
Course of the titration experiment of **1** with bis- TBA glutarate. • Δδ NH-pyrrole (10.11 to 13.68 ppm), ▪ Δδ NH-amide (7.23 to 9.91 ppm).

Plotting the shifts of one of the NH-protons of receptor **1** during titration with glutarate TBA salt (Figure 2), where the pyrrolic-NH and amide-NH moved 3.57 and 2.68 ppm downfield, respectively, afforded an association constant of K_ass_= 8.9×10^3^M^−1^ (see supplementary material). Table 1 shows the association constants obtained, including those found in studies carried out with the more rigid isophthalate and terephthalate TBA salts, which were chosen because their chain lengths are close to those of glutarate and adipate.

Both receptors show a higher K_ass_ to glutarate and adipate than to the other aliphatic bis-carboxylates studied, the K_ass_ of the dansyl receptor being slightly higher (8.9 and 8.2 × 10^3^ M^−1^ respectively) than those of the acridine receptor (5.8 and 3.9 × 10^3^ M^−1^ respectively). Lower K_ass_ values were obtained for succinate, which is conceivably too short to encompass the correct distance in these receptors, hence resulting on weaker binding. An interesting selectivity was found for glutarate as compared with succinate: about 35-fold and 28-fold higher with receptors **1** and **2**, respectively. Also, the rigidity of the isophthalate and terephthalate guests justifies the additional stabilization of their complexes, host **1** again showing higher of K_ass_ values than host **2**.

**Figure 3.**
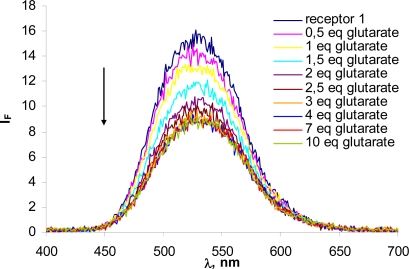
Decrease in fluorescence emission intensity observed for sensor **1**, c= 2.13×10^−5^ M in CH_3_Cl-DMSO(4%), upon the addition of the indicated increasing equivalents of bis-tetrabutylammonium glutarate: λ_ex_=350 nm, titrated with 0 → 10 eq.

To estimate the fluorescent response of receptors **1** and **2** towards bis-carboxylate anions, bis-TBA glutarate was selected. The quantum yield for fluorescence was assessed using quinine sulphate as standard, with the finding of Φ_F_ values of 0.09 for **1** and 0.01 for **2**. In order to determinate whether the receptors could be used as fluorescence-based sensors, we carried out absorption studies with UV-vis and fluorescence emission. Receptor **1** showed an absorption band centred at 354 nm (c= 2.13 × 10^−5^ M in chloroform-DMSO (4%) at 298 K) and no significant changes were observed in the absorption spectra during the addition of the glutarate salt (0–10 eq). When excited at a wavelength of 357 nm, **1** gave an emission maximum around 530 nm. On complexing with bis-TBA glutarate, a remarkable degree of fluorescence quenching was observed: about 20% upon addition of one equivalent and close to 50%, with respect to the receptor alone, when the saturation point was reached (Figure 3).

This system shows PET behaviour because of the complexing of glutarate will not change the aggregation state of the fluorophore but increase the reduction potential of amide-pyrrole receptor upon anion binding result in increased PET from the receptor to fluorophore and a quench in the fluorescence intensity. In order to ensure that the PET process was in fact operating, the concentration dependence of the change in fluorescence was checked, observing that the wavelengths of excitation and emission maxima remained invariant upon changing the bis-carboxylate concentrations, which is characteristic of a PET sensor [9,18,19]. In contrast, the acridine receptor **2** showed maximum absorption at 357 nm but very low fluorescence emission at 430 nm.

## 3. Conclusions

In conclusion, we have developed good neutral ditopic bis-2-pyrroleamide receptors **1** and **2** for binding of bis-carboxylates. Both receptors form 1:1 stoichiometric complexes with the guests and show moderate selectivity towards the carbon chain length of the bis-carboxylate anions. Of particular interest is the 35-fold selectivity obtained for glutarate over succinate. **1** shows interesting PET fluorescence activity in glutarate recognition.

## Supplementary material:



## Figures and Tables

**Scheme I.**
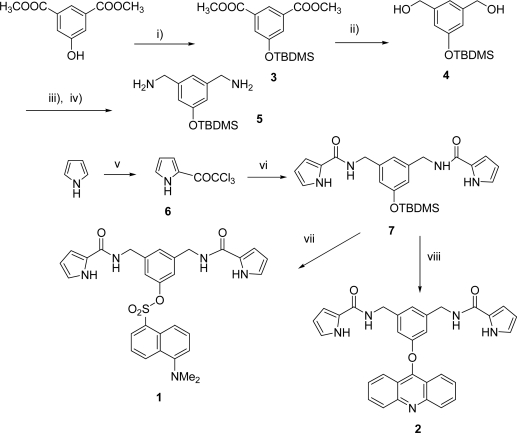
Synthesis of receptors **1** and **2**. i) *t*-BuMe_2_SiCl, imidazole, DMF, 97%; ii) LiAlH_4_, diethyl ether, 95%; iii) DIAD, PPh_3_, phthalimide, THF and iv) N_2_H_4_, EtOH, 44%; v) CCl_3_COCl, lutidine, CHCl_3_, 80%; vi) **5**, CH_2_Cl_2_, 70%; vii) TBAF 1M in THF, dansyl chloride, CH_2_Cl_2_/THF, 76%; viii) TBAF 1M in THF, 9-chloroacridine, CH_2_Cl_2_/THF, 92%

**Table 1.** Association constants of receptors **1** and **2** with bis-TBA salts of bis-carboxylate anions in CDCl_3_/DMSO-d_6_ (4%). In all cases 1:1 receptor:anion stoichiometry was observed.

**Guest**	**1 K_ass_ (M^−1^)**	**2 K_ass_ (M^−1^)**
Succinate	2.5×10^2^	2.1×10^2^
Glutarate	8.9×10^3^	5.8×10^3^
Adipate	8.2×10^3^	3.9×10^3^
Pimelate	5.3×10^3^	3.1×10^3^
Suberate	4.0×10^3^	2.8×10^3^
Isophthalate	4.2×10^4^	1.7 ×10^4^
Terephthalate	1.0×10^4^	6.2×10^3^
