# Supplementary material for: Synthesis, Binding and Fluorescence Studies of Bis-2-amidopyrrole Receptors for Bis-carboxylate Anions
Source: Sensors (Basel). 2009 Mar 4;9(3):1534–40. doi: 10.3390/s90301534 (PMC3345866; doi:10.3390/s90301534)
Supplement: Supplementary file 1 [file sensors-09-01534-s001.pdf]

Supplementary material:

1.- ^{1}H and ^{13}C-NMR spectra of **1** and **2** ………………………… p. 2

2.- HRMS of **1** and **2** …………………………………………….……… p. 7

3a.- Titration spectra of **1** with dicarboxylates …………………..… p. 11

3b.- Titration spectra of **2** with dicarboxylates …………………..… p. 17

4a.- Titration curves of **1** …………………………………………...… p. 23

4b.- Titration curves of **2** ………………………………………….… p. 31

5.- Fluorescence experiments of **1** with glutarate TBA-salt ……. p. 39

1.- ^{1}H and ^{13}C-NMR spectra of **1** and **2**

belen-20 receptor 4
1H DMSO-d6

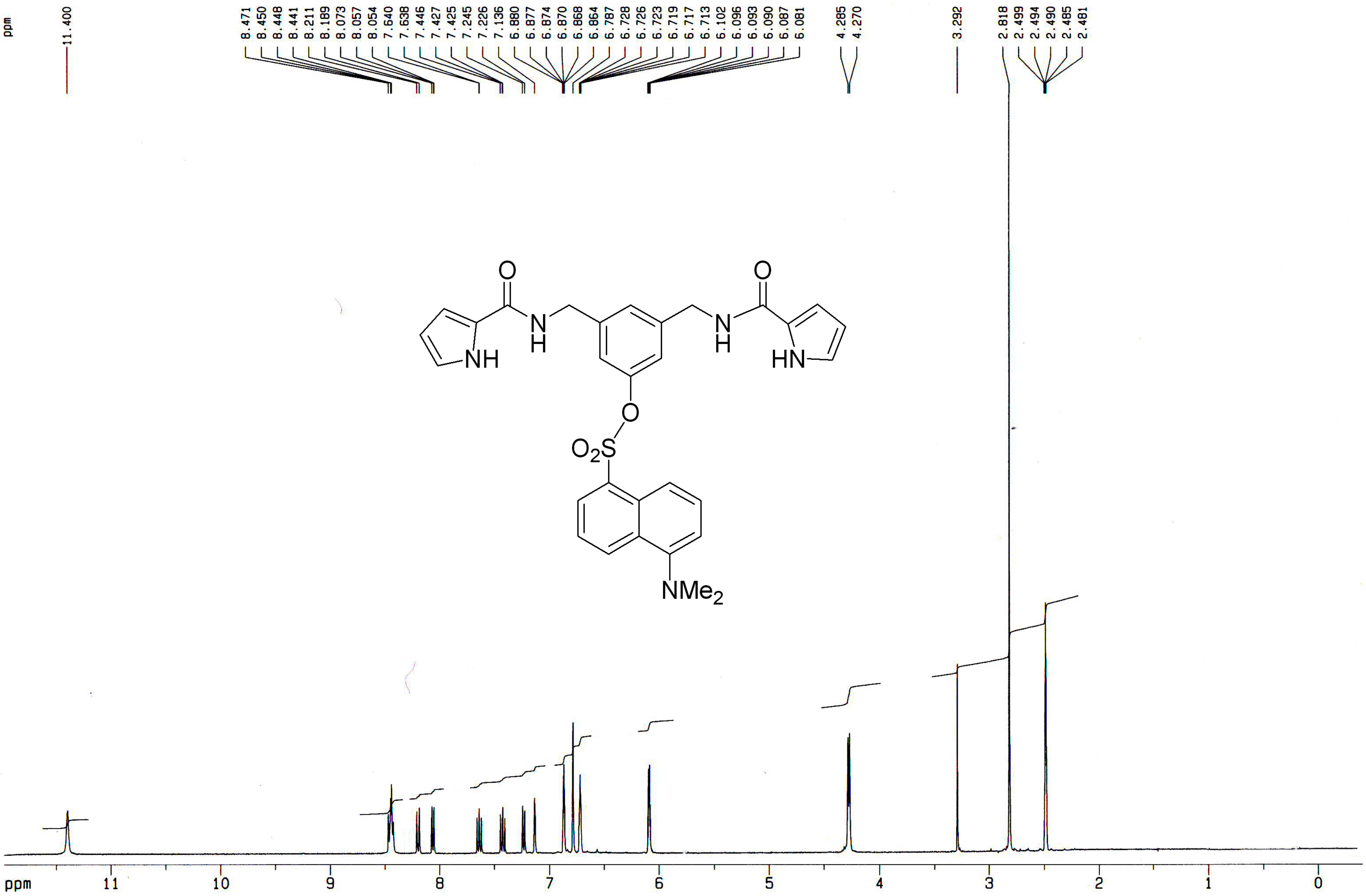

belen-19 receptor 3
1H DMSO-d6

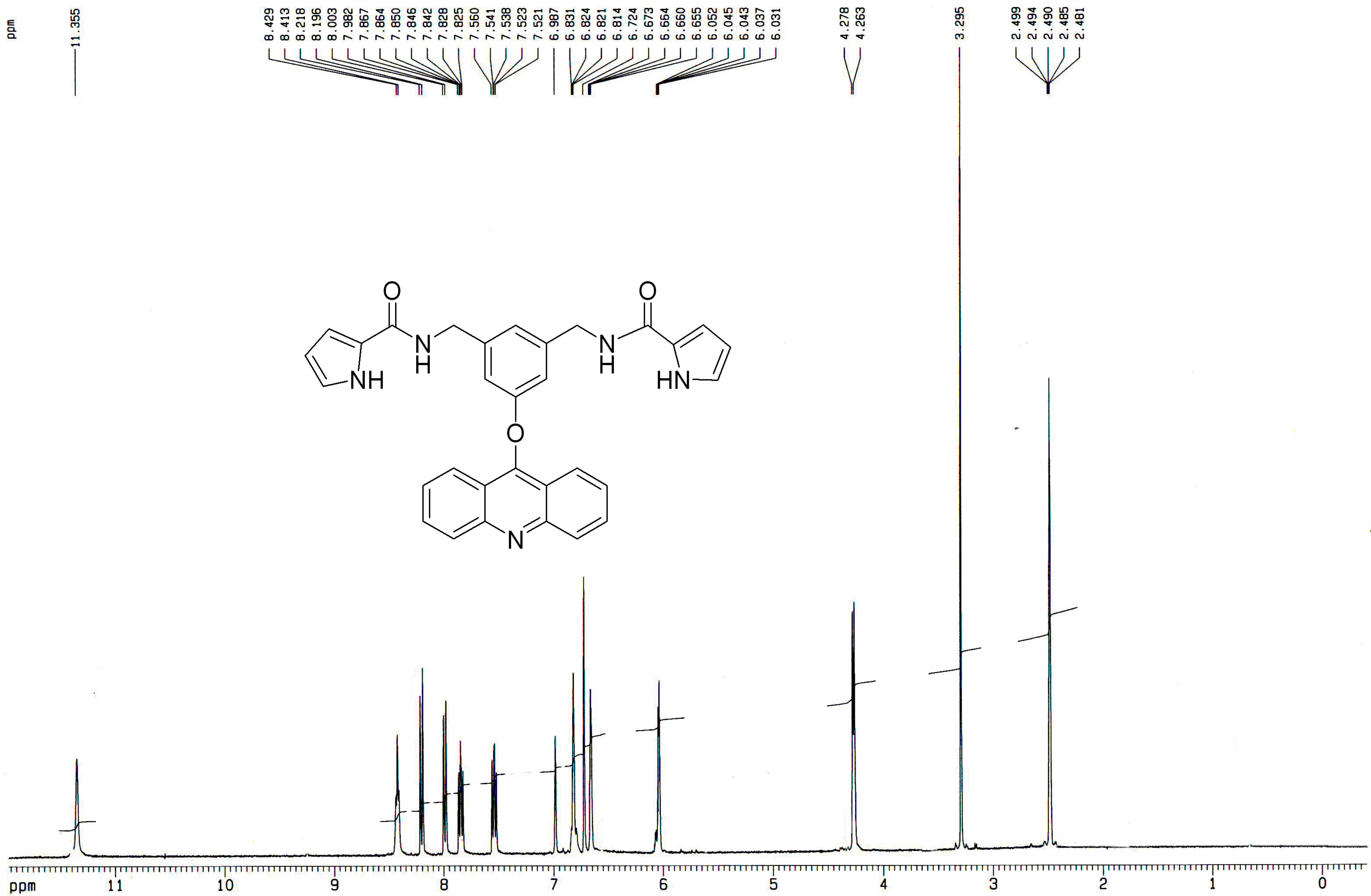

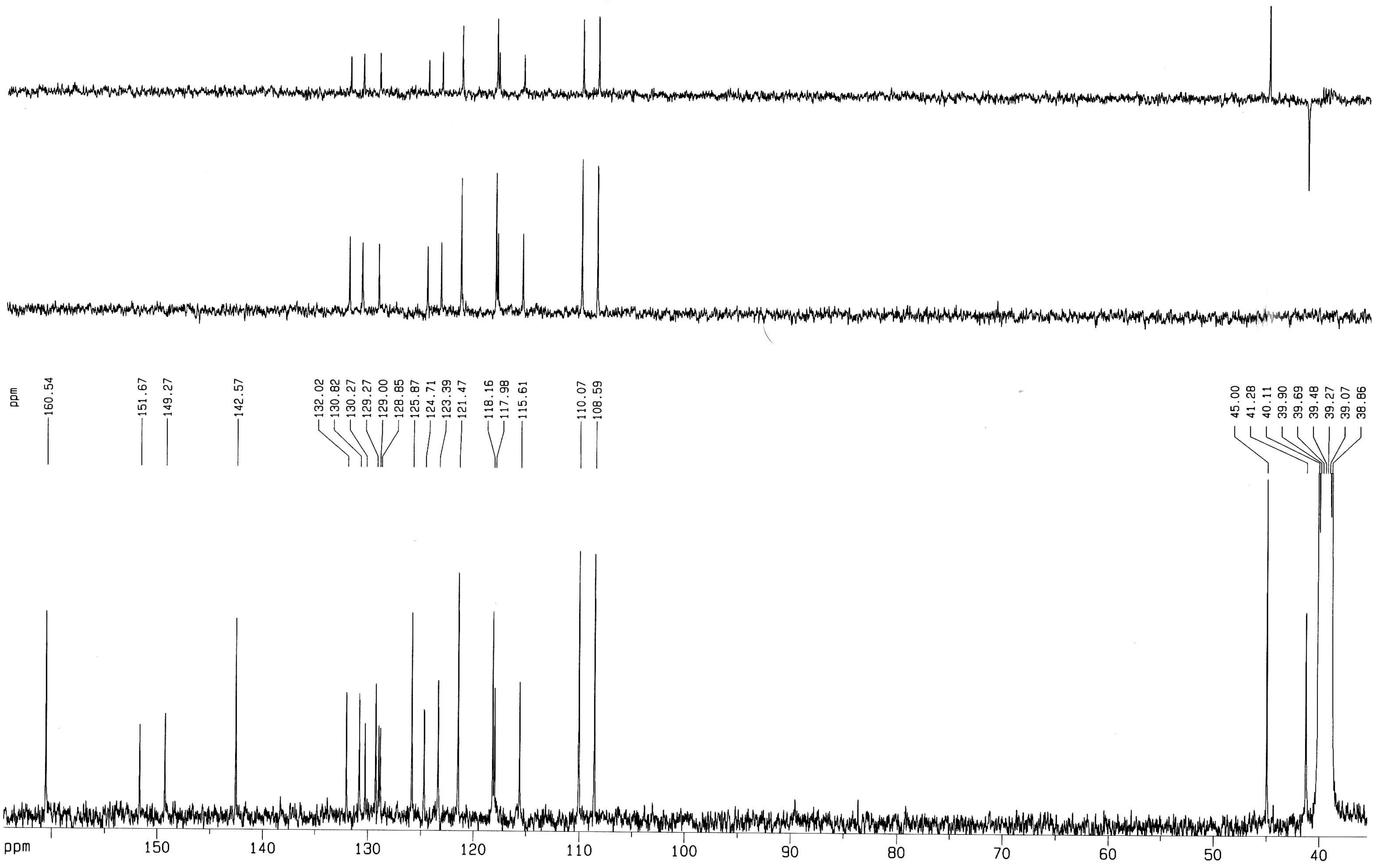
ppm
160.54
151.67
149.27
142.57
132.02
130.82
130.27
129.27
129.00
128.85
125.87
124.71
123.39
121.47
118.16
117.98
115.61
110.07
108.59
45.00
41.28
40.11
39.90
39.69
39.48
39.27
39.07
38.86
ppm
150
140
130
120
110
100
90
80
70
60
50
40

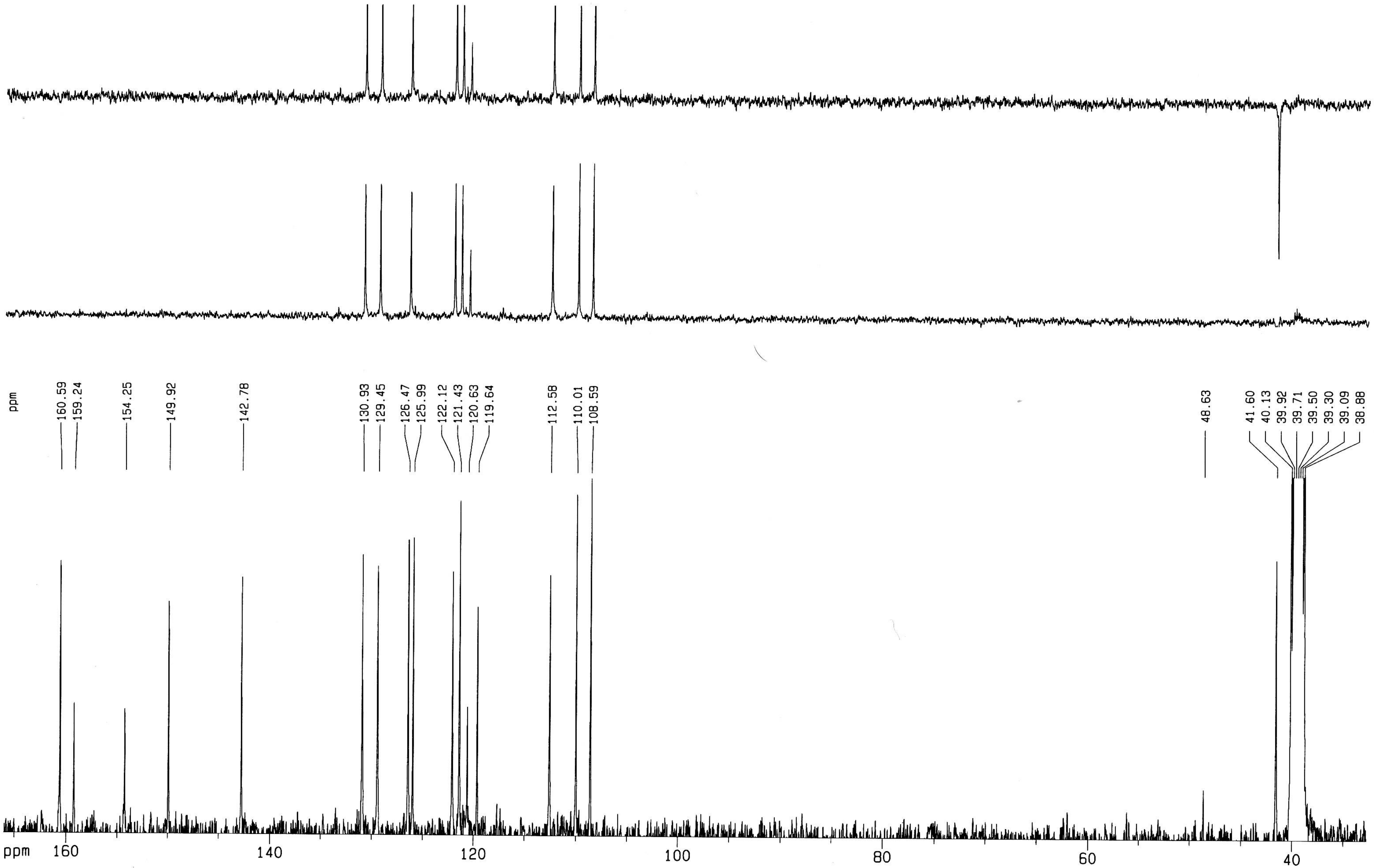
ppm
160.59
159.24
154.25
149.92
142.78
130.93
129.45
126.47
125.99
122.12
121.43
120.63
119.64
112.58
110.01
108.59
48.63
41.60
40.13
39.92
39.71
39.50
39.30
39.09
38.88
ppm 160 140 120 100 80 60 40

2.- HRMS of **1** and **2**

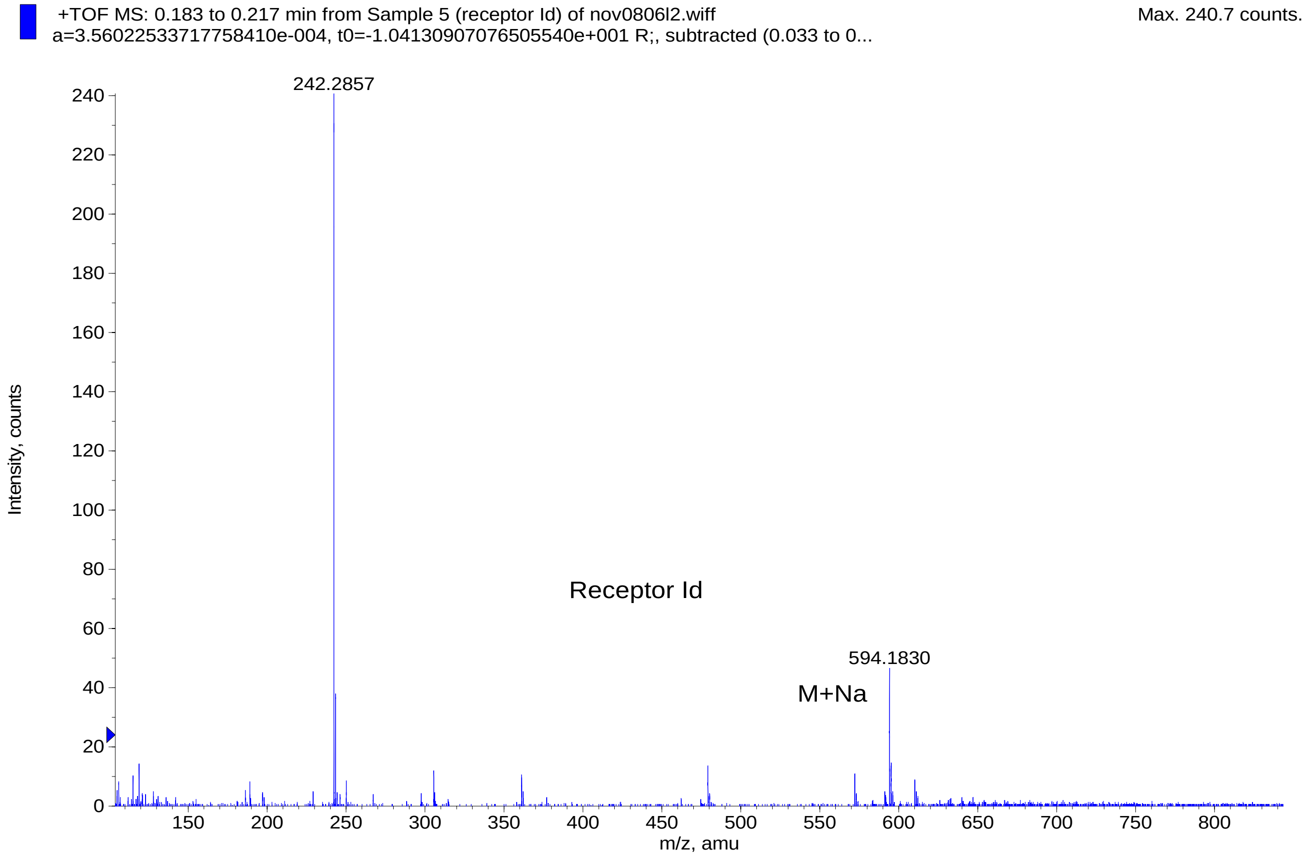

Formula	CalculatedMass	mDaError	ppmError	RDB
C23 H33 N5 O10 Na S	594.184036	-1.03568	-1.74303	9.5
C32 H28 N5 O5 S	594.180568	2.43242	4.093718	21.5
C25 H32 N5 O10 S	594.186441	-3.44094	-5.791039	12.5
C30 H29 N5 O5 Na S	594.178162	4.83768	8.141727	18.5

+TOF MS: 0.167 to 0.200 min from Sample 4 (receptor Ia) of nov0806l2.wiff Max. 1757.7 counts.
a=3.56022533717758410e-004, t0=-1.04130907076505540e+001 R;, subtracted (0.034 to ...

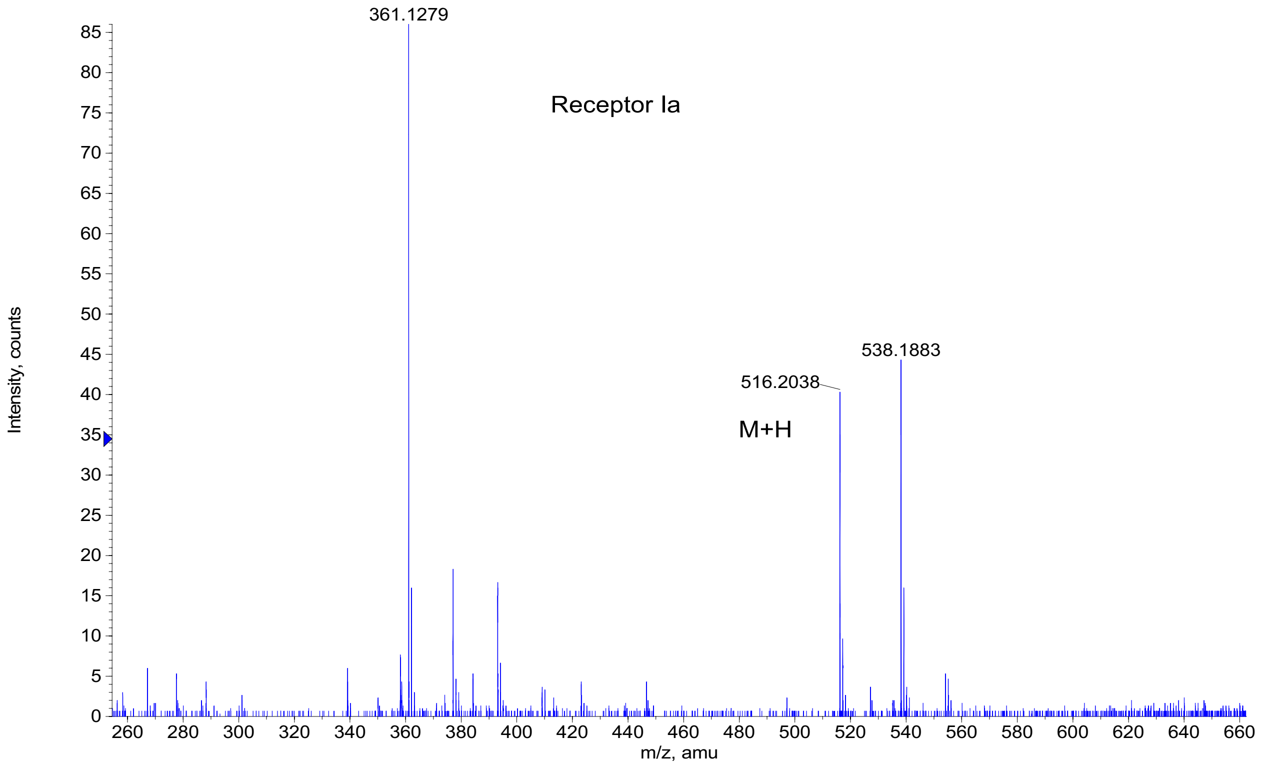

Formula	CalculatedMass	mDaError	ppmError	RDB
C18 H27 N11 O6 Na	516.203799	0.000872	0.001689	10.5
C25 H31 N5 O4 Na Si	516.203754	0.04564	0.088415	13.5
C10 H34 N9 O13 Si	516.203988	-0.187572	-0.363368	-0.5
C18 H34 N3 O14	516.20353	0.270236	0.523506	3.5
C23 H26 N11 O2 Si	516.203474	0.325772	0.631091	17.5
C31 H26 N5 O3	516.203016	0.78358	1.517965	21.5
C34 H27 N3 O Na	516.204634	-0.833864	-1.615376	22.5
C9 H31 N13 O9 Na Si	516.20292	0.880376	1.70548	1.5
C26 H34 N O8 Si	516.204822	-1.022308	-1.980433	11.5
C19 H30 N7 O10	516.204867	-1.067076	-2.067158	8.5
C26 H27 N9 Na Si	516.205092	-1.291672	-2.502249	18.5
C19 H23 N15 O2 Na	516.205136	-1.33644	-2.588975	15.5
C17 H31 N7 O10 Na	516.202462	1.338184	2.592353	5.5
C21 H35 N O12 Na	516.205147	-1.347208	-2.609835	4.5
C24 H35 N O8 Na Si	516.202417	1.382952	2.679079	8.5
C11 H30 N13 O9 Si	516.205325	-1.524884	-2.954032	4.5
C15 H26 N13 O8	516.202182	1.618316	3.13503	9.5
C22 H30 N7 O6 Si	516.202137	1.663084	3.221755	12.5
C13 H35 N7 O11 Na Si	516.205605	-1.805016	-3.496708	0.5
C30 H30 N O7	516.201679	2.120892	4.108629	16.5
C27 H30 N5 O4 Si	516.20616	-2.35962	-4.571097	16.5
C20 H26 N11 O6	516.206204	-2.404388	-4.657822	13.5
C6 H30 N15 O11 Si	516.201302	2.49782	4.83882	0.5

C16 H35 N3 O14 Na	516.201125	2.675496	5.183017	0.5
C22 H31 N5 O8 Na	516.206485	-2.68452	-5.200499	9.5
C21 H27 N11 O2 Na Si	516.201069	2.731032	5.290602	14.5
C14 H30 N9 O12	516.200844	2.955628	5.725694	4.5

3 a.- Titration spectra of **1** with dicarboxylates

3.1. Succinate

3.2. Adipate

3.3. Pimelate

3.4. Isophthalate

3.5. Terphthalate

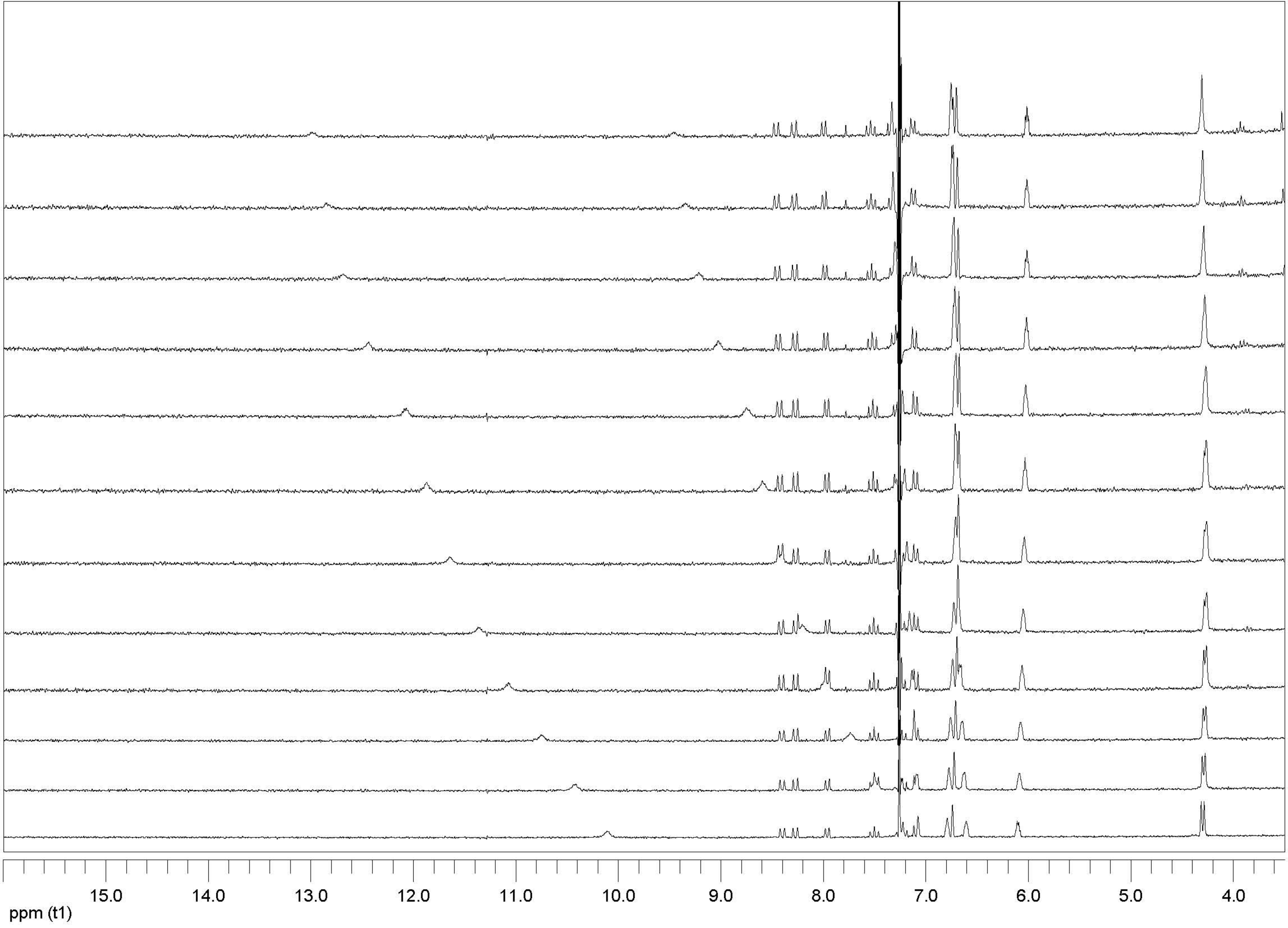
15.0
14.0
13.0
12.0
11.0
10.0
9.0
8.0
7.0
6.0
5.0
4.0
ppm (t1)

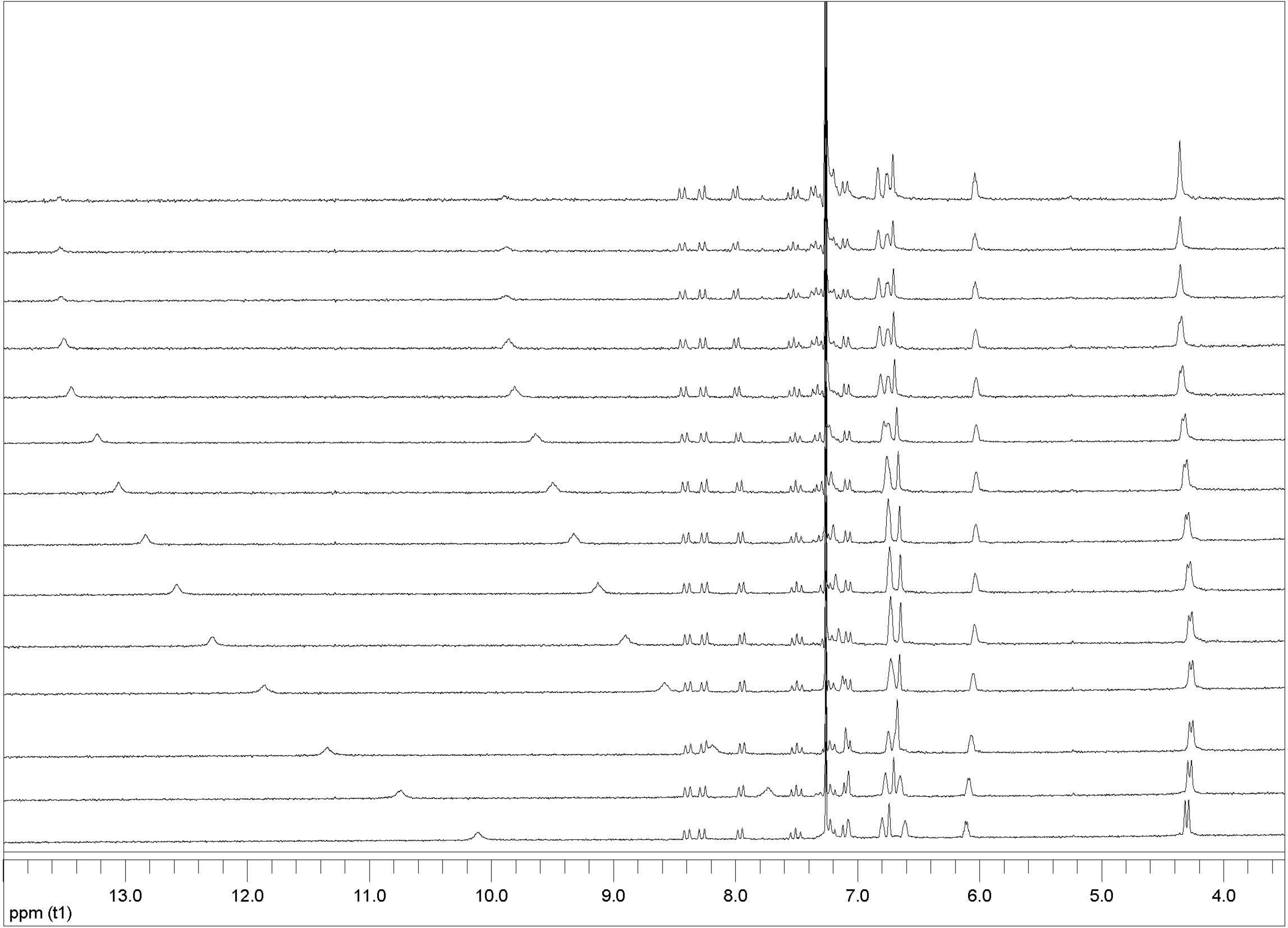

13.0
12.0
11.0
10.0
9.0
8.0
7.0
6.0
5.0
4.0
ppm (t1)

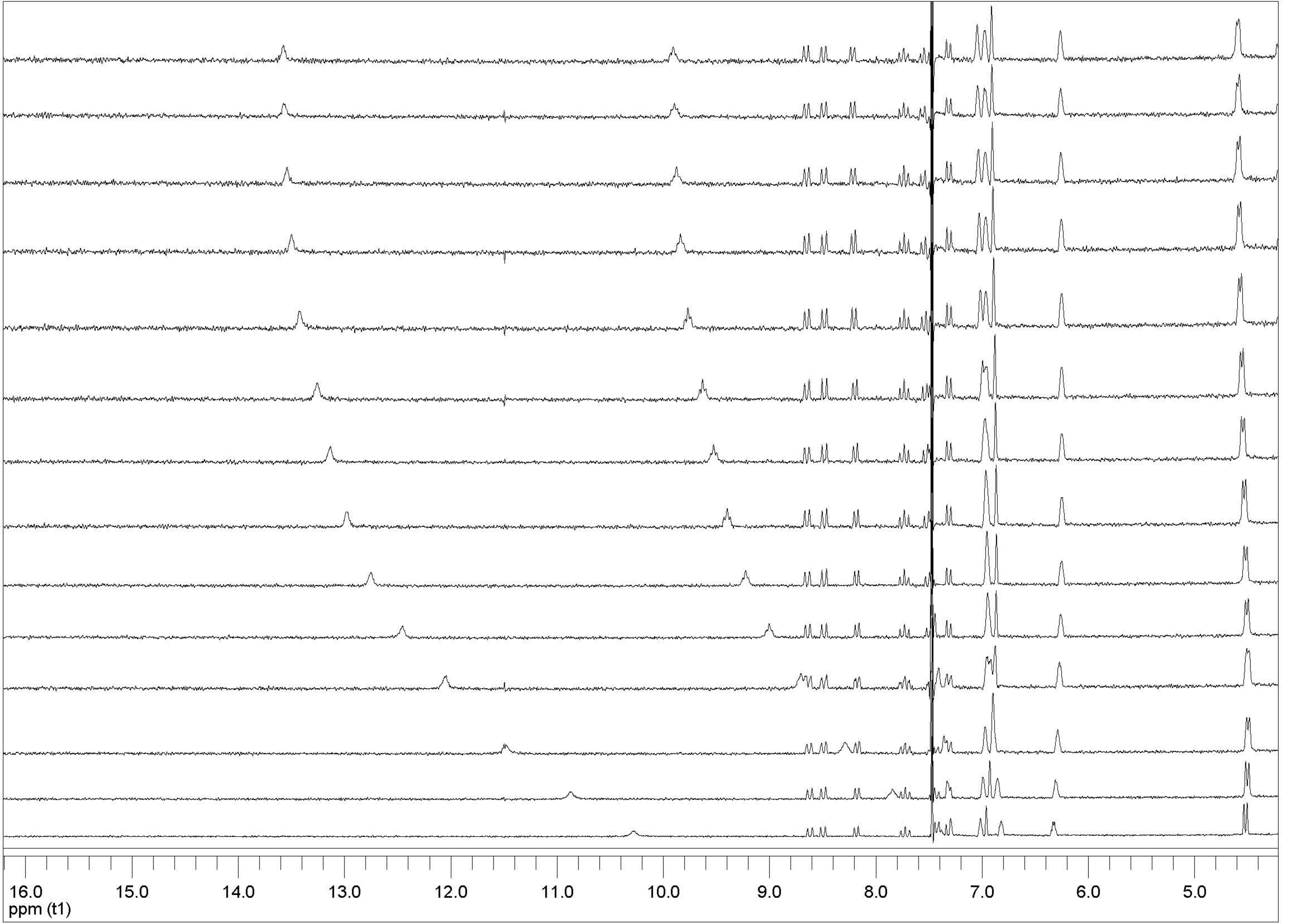

16.0
15.0
14.0
13.0
12.0
11.0
10.0
9.0
8.0
7.0
6.0
5.0
ppm (t1)

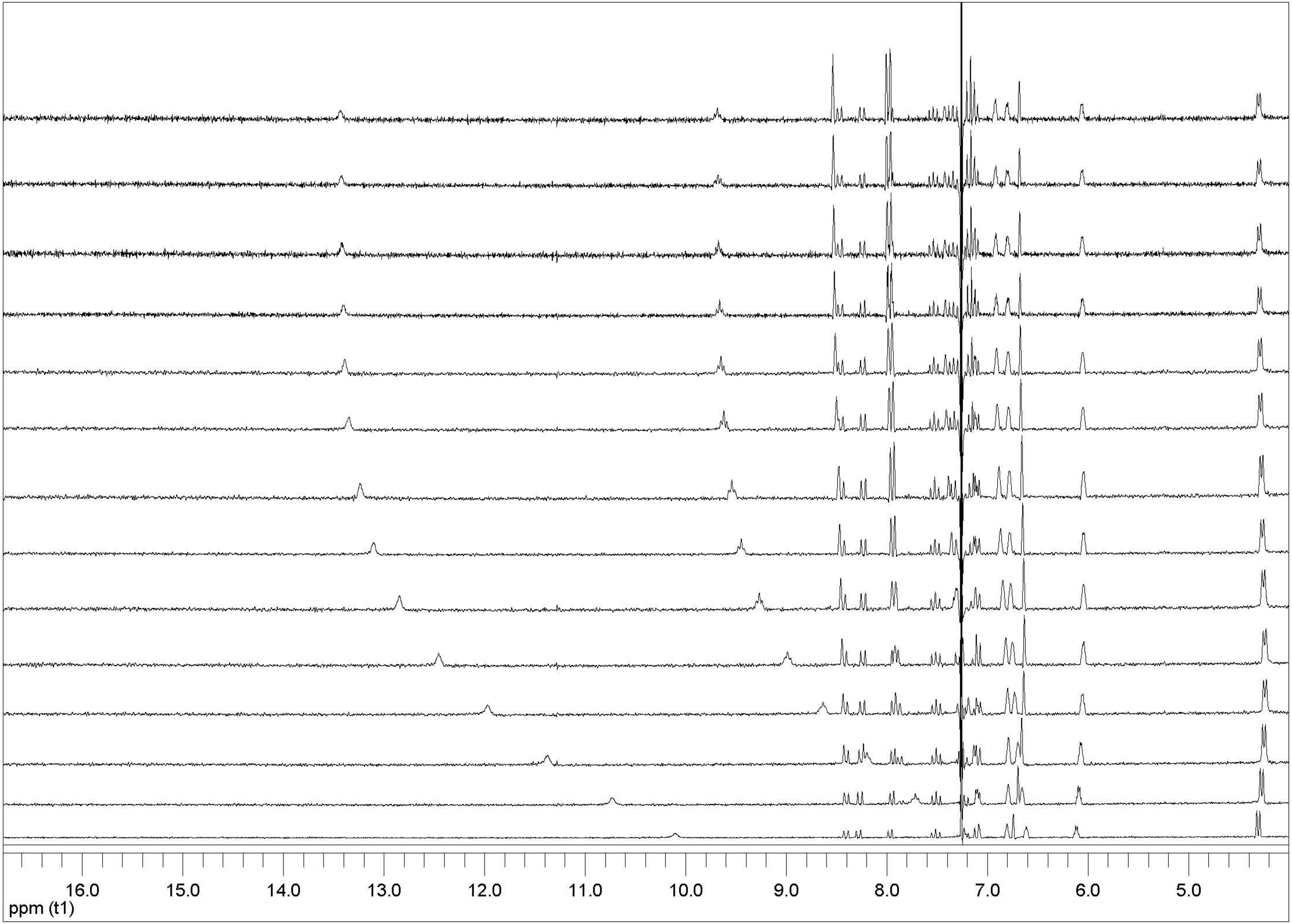
16.0
15.0
14.0
13.0
12.0
11.0
10.0
9.0
8.0
7.0
6.0
5.0
ppm (t1)

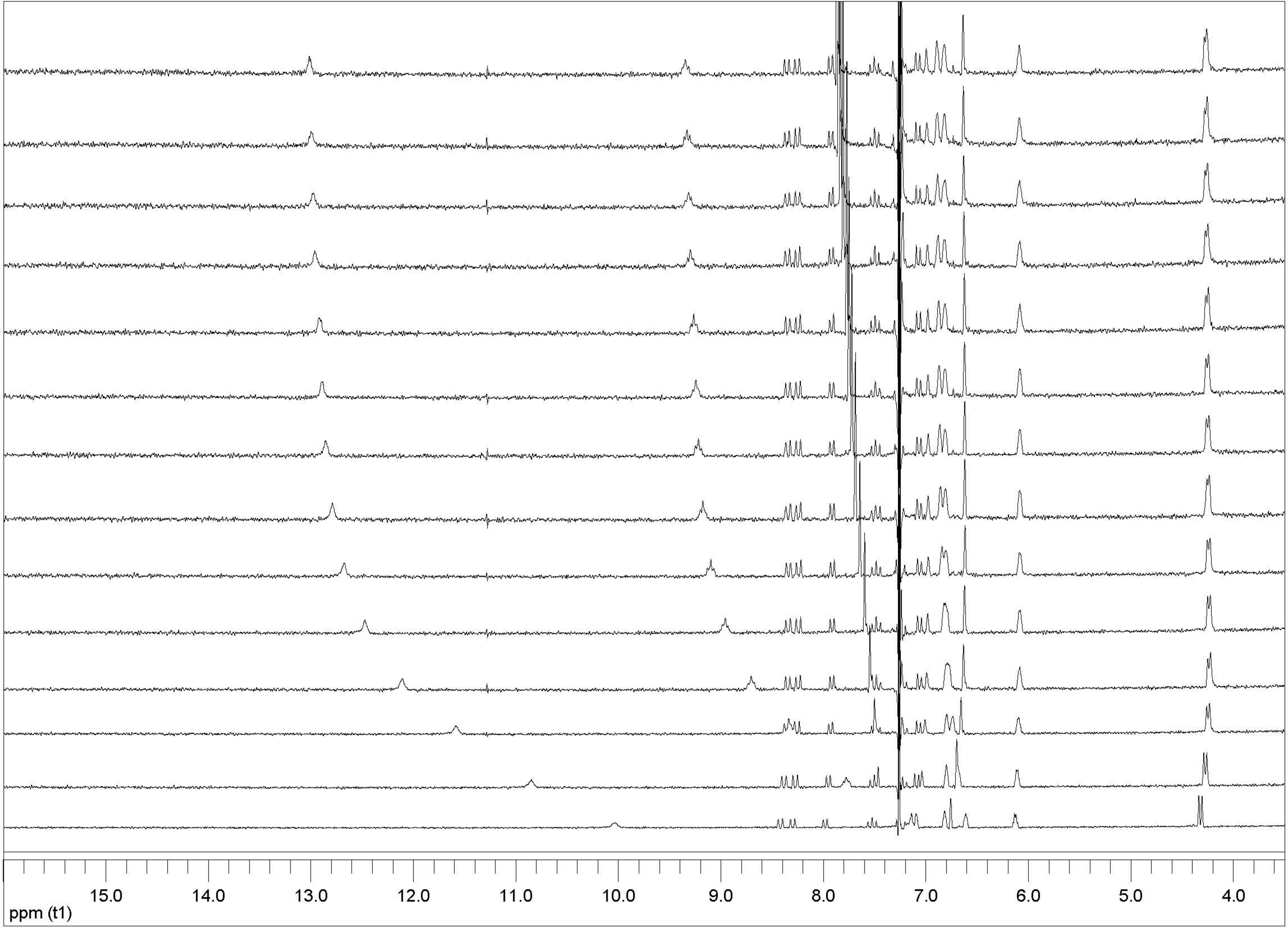
15.0
14.0
13.0
12.0
11.0
10.0
9.0
8.0
7.0
6.0
5.0
4.0
ppm (t1)

3 b.- Titration spectra of **2** with dicarboxylates

3.1. Succinate

3.2. Glutarate

3.3. Pimelate

3.4. Isophthalate

3.5. Terphthalate

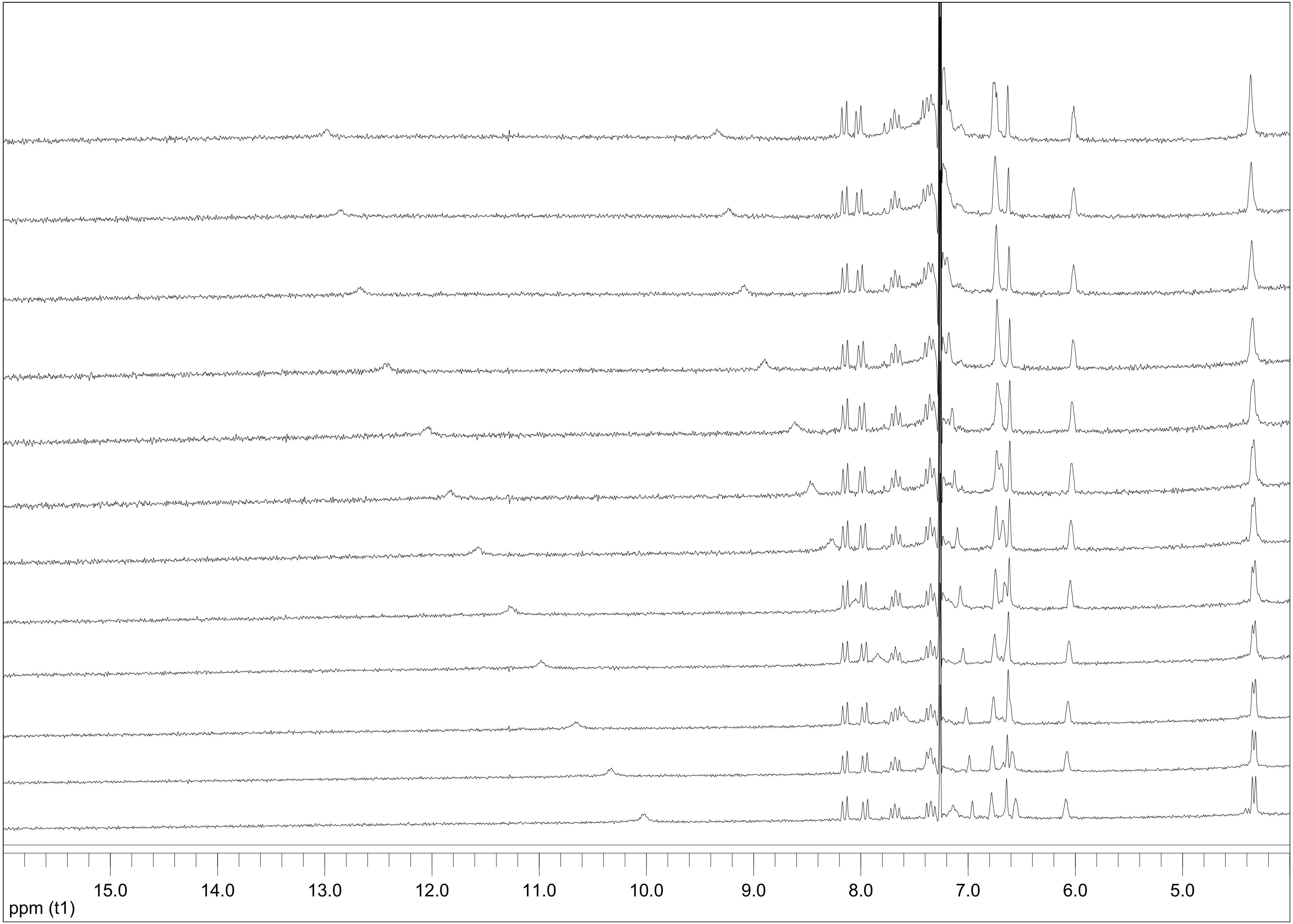
15.0
14.0
13.0
12.0
11.0
10.0
9.0
8.0
7.0
6.0
5.0
ppm (t1)

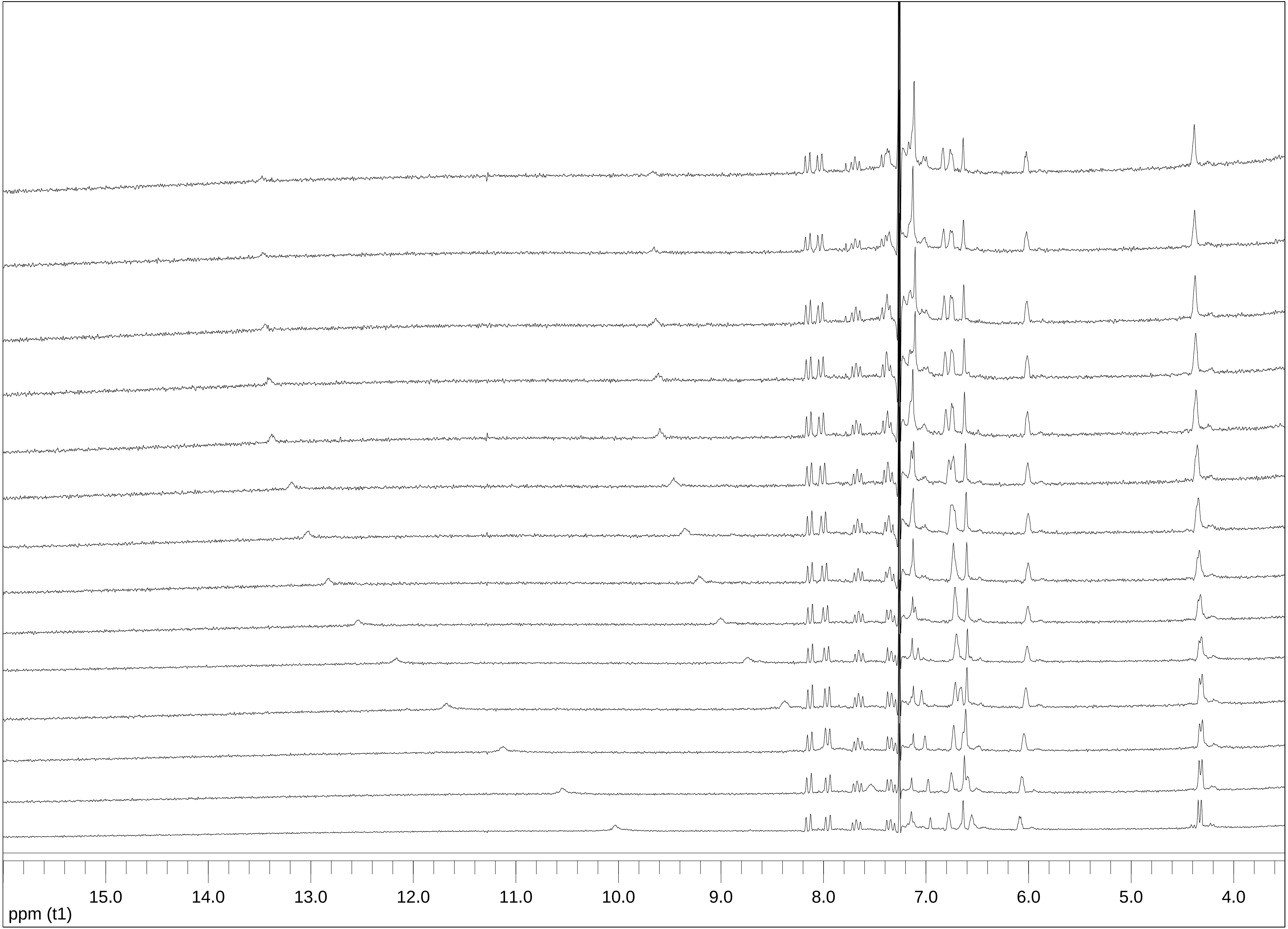
15.0
14.0
13.0
12.0
11.0
10.0
9.0
8.0
7.0
6.0
5.0
4.0
ppm (t1)

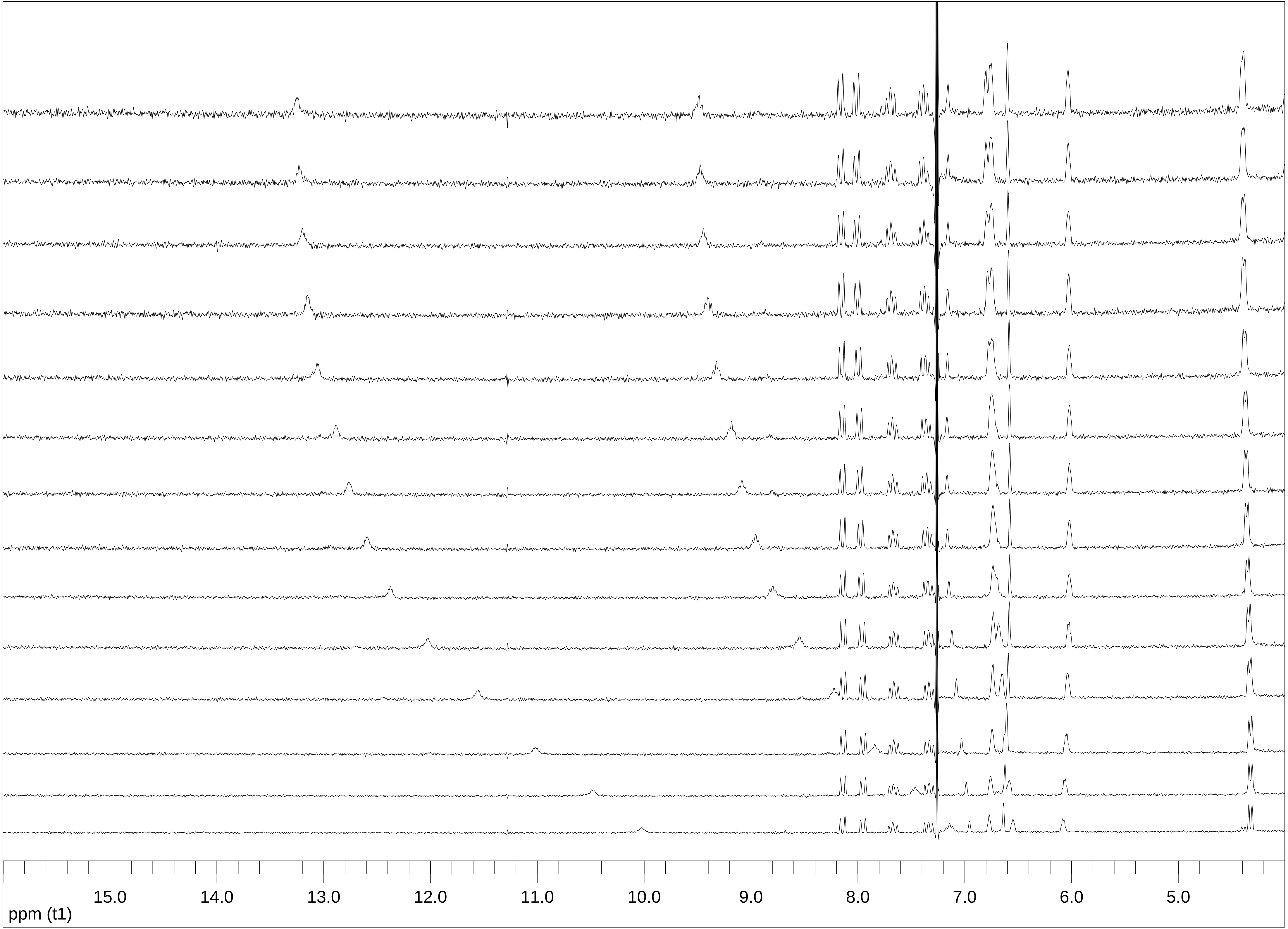
15.0
14.0
13.0
12.0
11.0
10.0
9.0
8.0
7.0
6.0
5.0
ppm (t1)

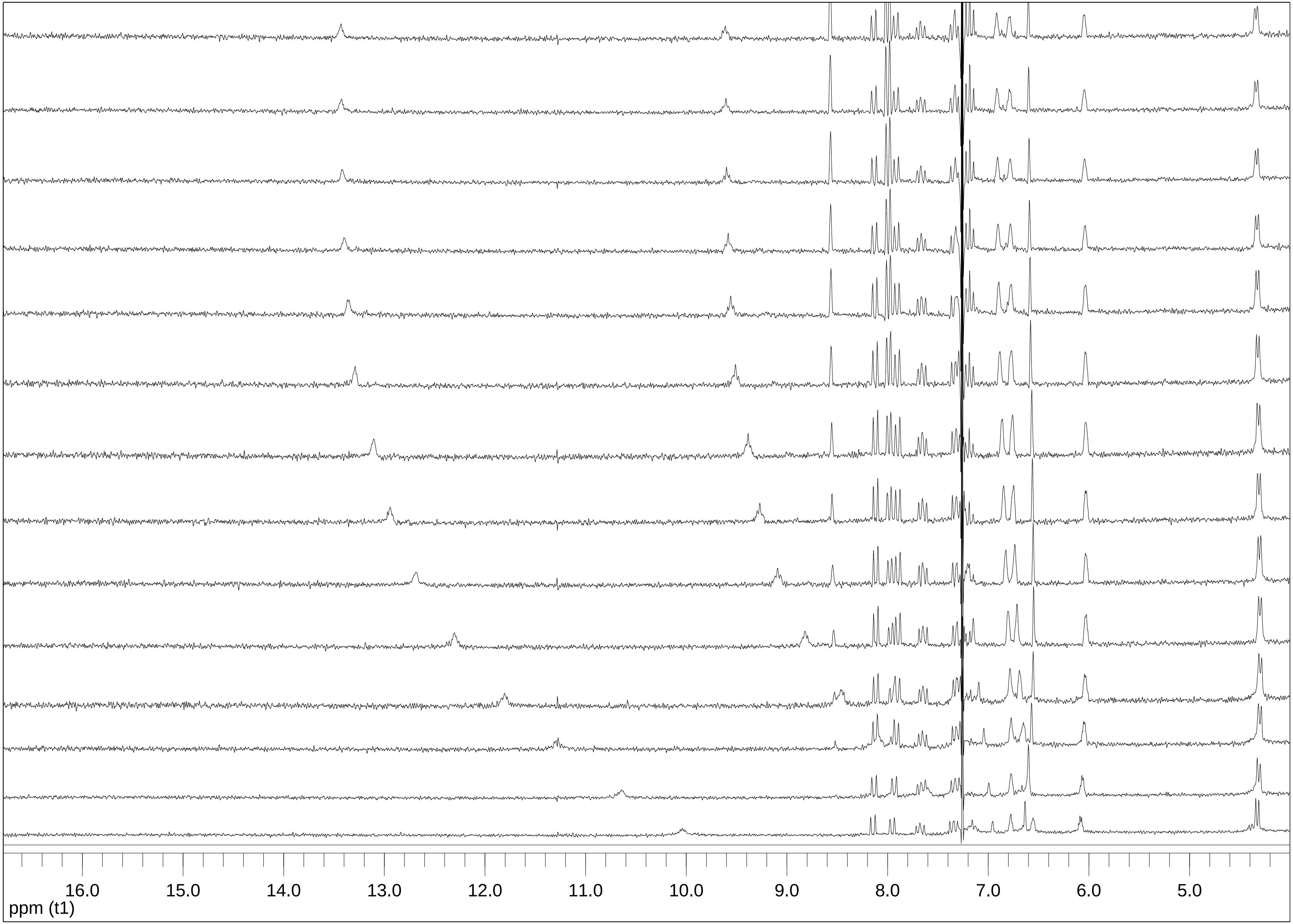

16.0
15.0
14.0
13.0
12.0
11.0
10.0
9.0
8.0
7.0
6.0
5.0
ppm (t1)

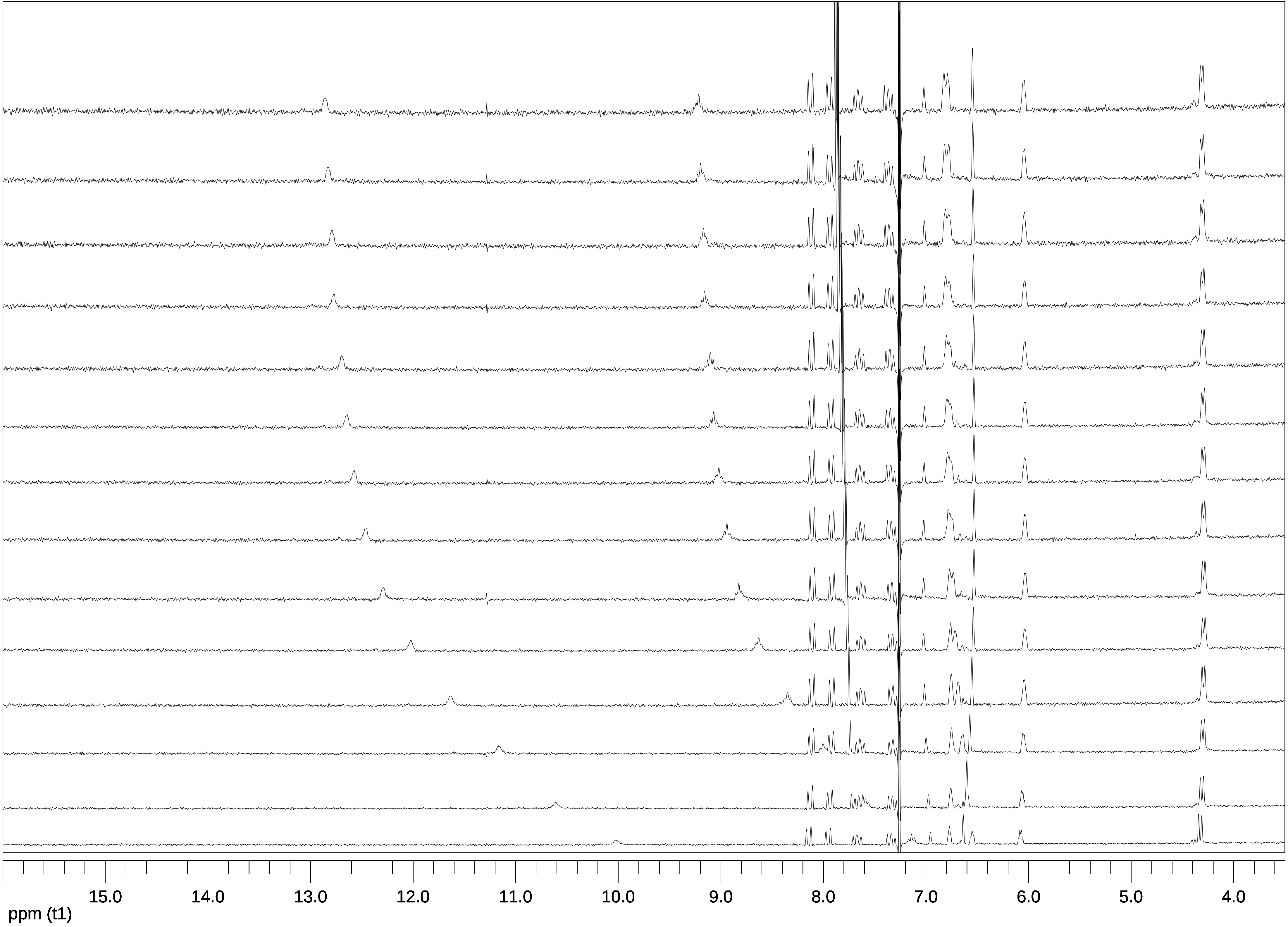
15.0
14.0
13.0
12.0
11.0
10.0
9.0
8.0
7.0
6.0
5.0
4.0
ppm (t1)

4 a.- Titration curves of **1** with:

4.1. Succinate

4.2. Glutarate

4.3. Adipate

4.4. Pimelate

4.5. Suberate

4.6. Isophthalate

4.7. Terphthalate

Constante de Asociación
Constante: 2.5e+02 1/M
Despl. Max.: 15.191

Host: receptor Id
Peso molecular: 572
Peso puesto (mg): 1.2
Volumen total (ml): 0.5
Volumen puesto (ml): 0.5

Guest: succinato cloroformo-DMSO
Peso molecular: 600
Peso puesto(mg): 10.6
Volumen puesto(ml): 1

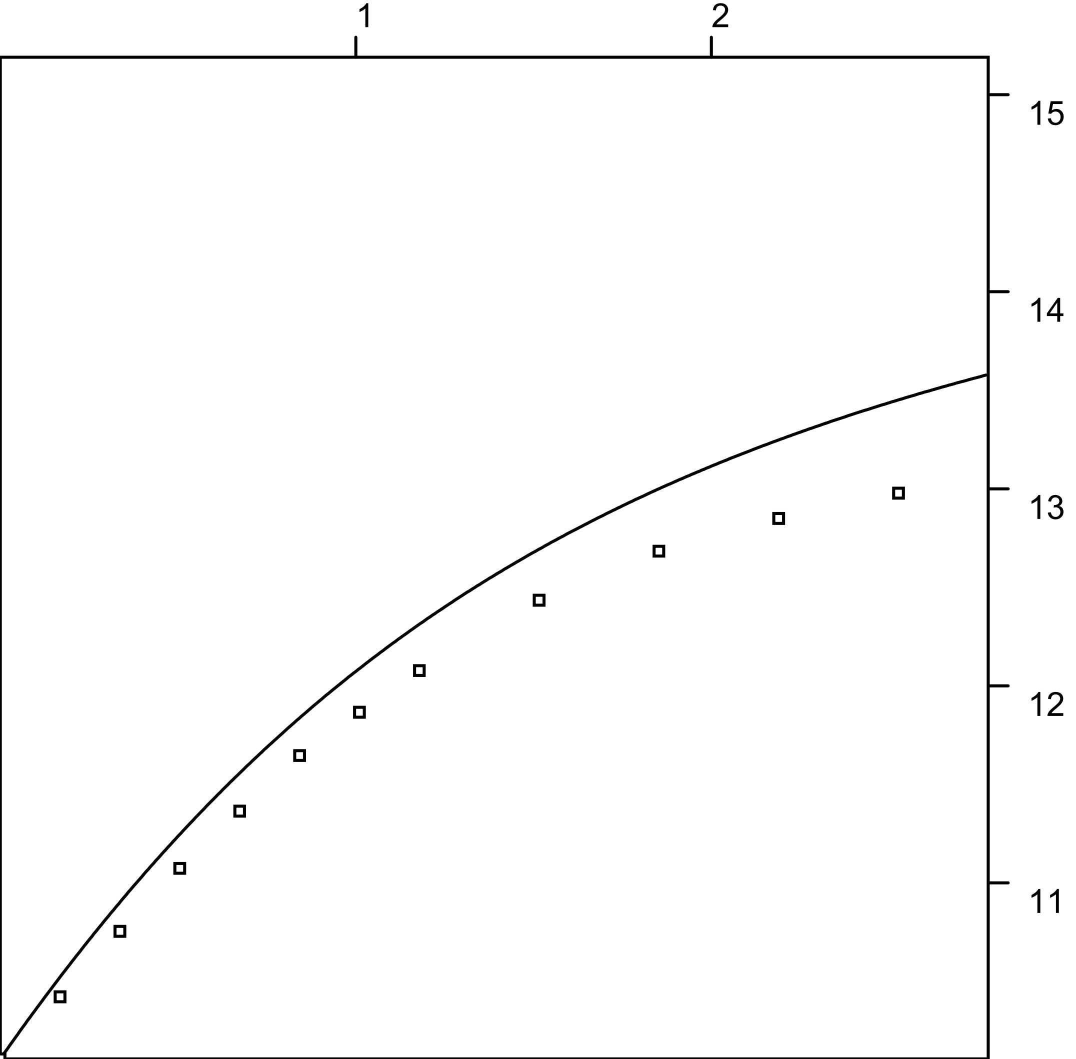

Desplazamiento	Volumen
10.106	0
10.421	0.02
10.753	0.02
11.073	0.02
11.363	0.02
11.646	0.02
11.866	0.02
12.077	0.02
12.434	0.04
12.683	0.04
12.849	0.04
12.977	0.04

Constante de Asociación
Constante: 8.9e+03 1/M
Despl. Max.: 13.826

Host: Id
Peso molecular: 572
Peso puesto (mg): 1.26
Volumen total (ml): 0.5
Volumen puesto (ml): 0.5

Guest: Glutarato
Peso molecular: 614
Peso puesto(mg): 9.8
Volumen puesto(ml): 1

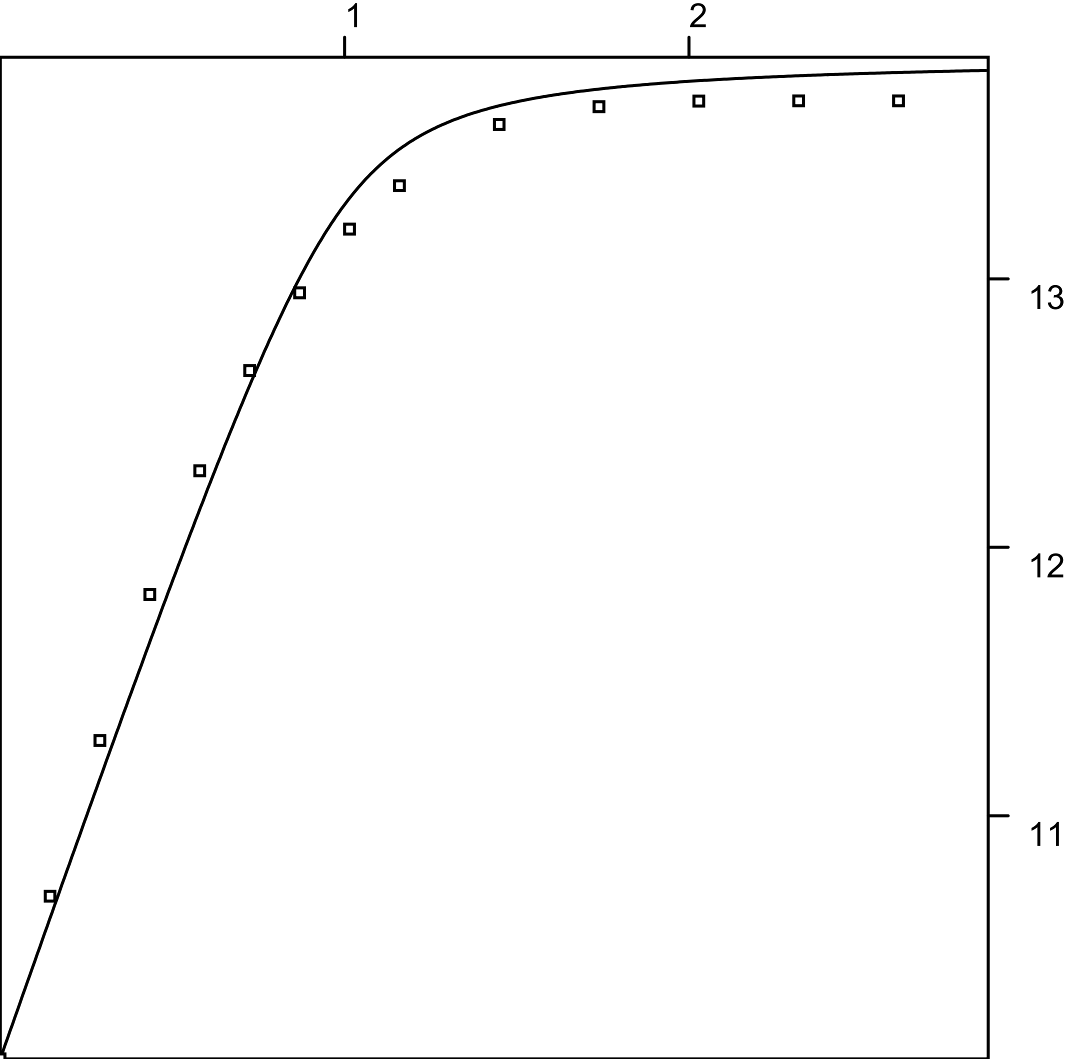

Desplazamiento	Volumen
10.094	0
10.7	0.02
11.28	0.02
11.824	0.02
12.285	0.02
12.658	0.02
12.948	0.02
13.185	0.02
13.347	0.02
13.575	0.04
13.641	0.04
13.662	0.04
13.663	0.04
13.663	0.04

Constante de Asociación
Constante: 5.6e+03 1/M
Despl. Max.: 13.739

Host: Id
Peso molecular: 572
Peso puesto (mg): 1.14
Volumen total (ml): 0.5
Volumen puesto (ml): 0.5

Guest: Adipato
Peso molecular: 628
Peso puesto(mg): 9.9
Volumen puesto(ml): 1

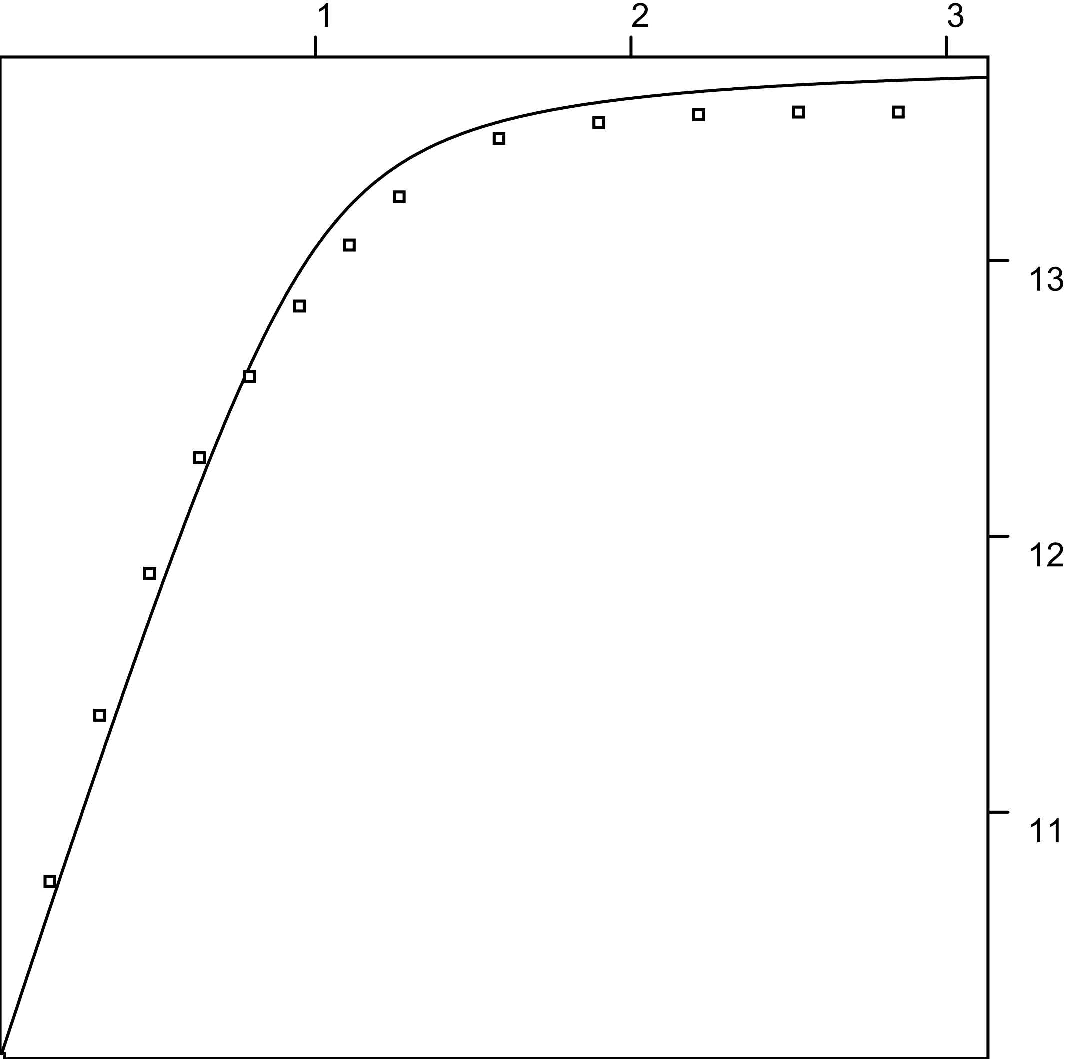

Desplazamiento	Volumen
10.106	0
10.749	0.02
11.351	0.02
11.866	0.02
12.285	0.02
12.579	0.02
12.835	0.02
13.056	0.02
13.231	0.02
13.442	0.04
13.5	0.04
13.529	0.04
13.538	0.04
13.538	0.04

Constante de Asociación
Constante: 5.3e+03 1/M
Despl. Max.: 9.8159

Host: receptor Id
Peso molecular: 572
Peso puesto (mg): 1.26
Volumen total (ml): 0.5
Volumen puesto (ml): 0.5

Guest: pimelato cloroformo-DMSO
Peso molecular: 644
Peso puesto(mg): 11.1
Volumen puesto(ml): 1

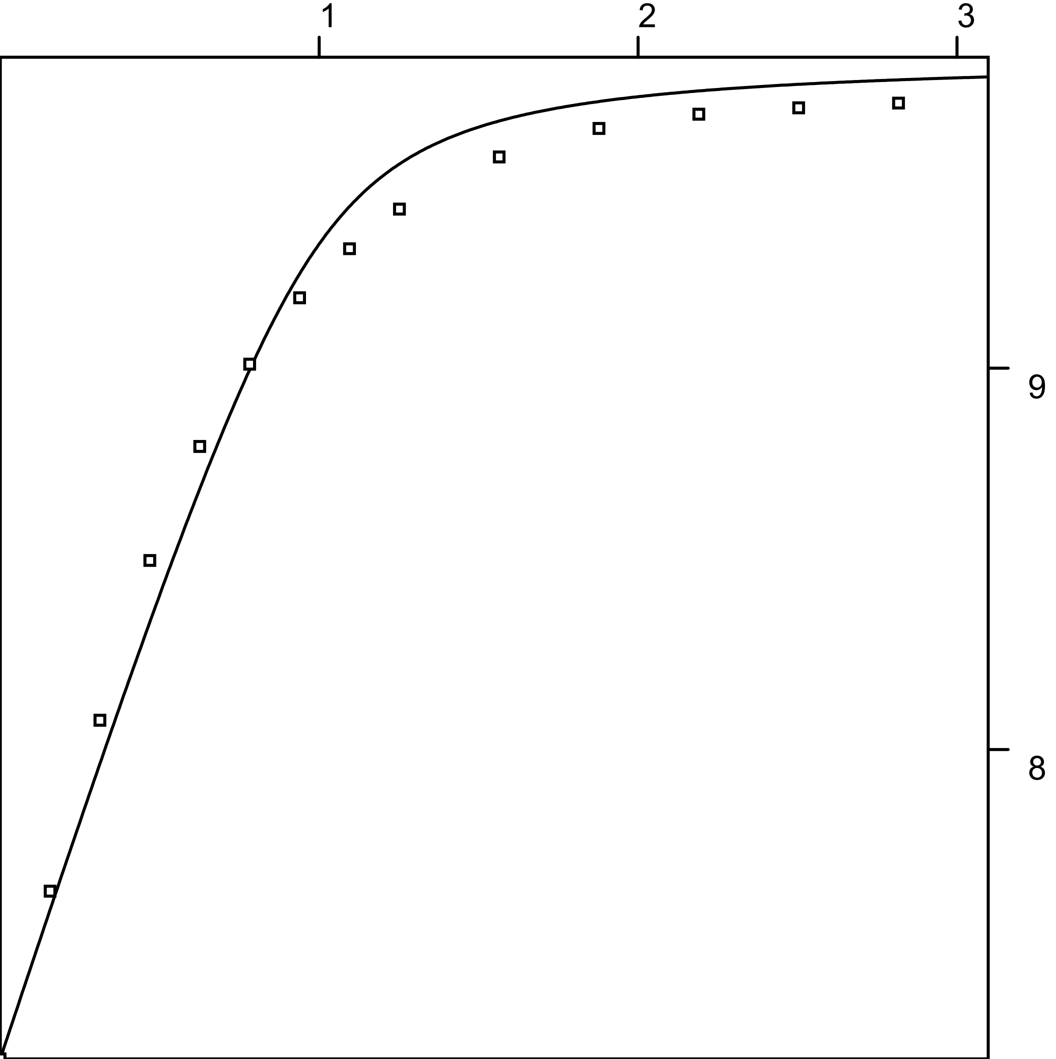

Desplazamiento	Volumen
7.1887	0
7.6285	0.02
8.0766	0.02
8.4957	0.02
8.7945	0.02
9.0102	0.02
9.1845	0.02
9.3131	0.02
9.4169	0.02
9.5538	0.04
9.6285	0.04
9.6658	0.04
9.6824	0.04
9.6949	0.04

Constante de Asociación
Constante: 4e+03 1/M
Despl. Max.: 9.8952

Host: receptor Id
Peso molecular: 572
Peso puesto (mg): 1.14
Volumen total (ml): 0.5
Volumen puesto (ml): 0.5

Guest: suberato cloroformo-DMSO
Peso molecular: 656
Peso puesto(mg): 11.4
Volumen puesto(ml): 1

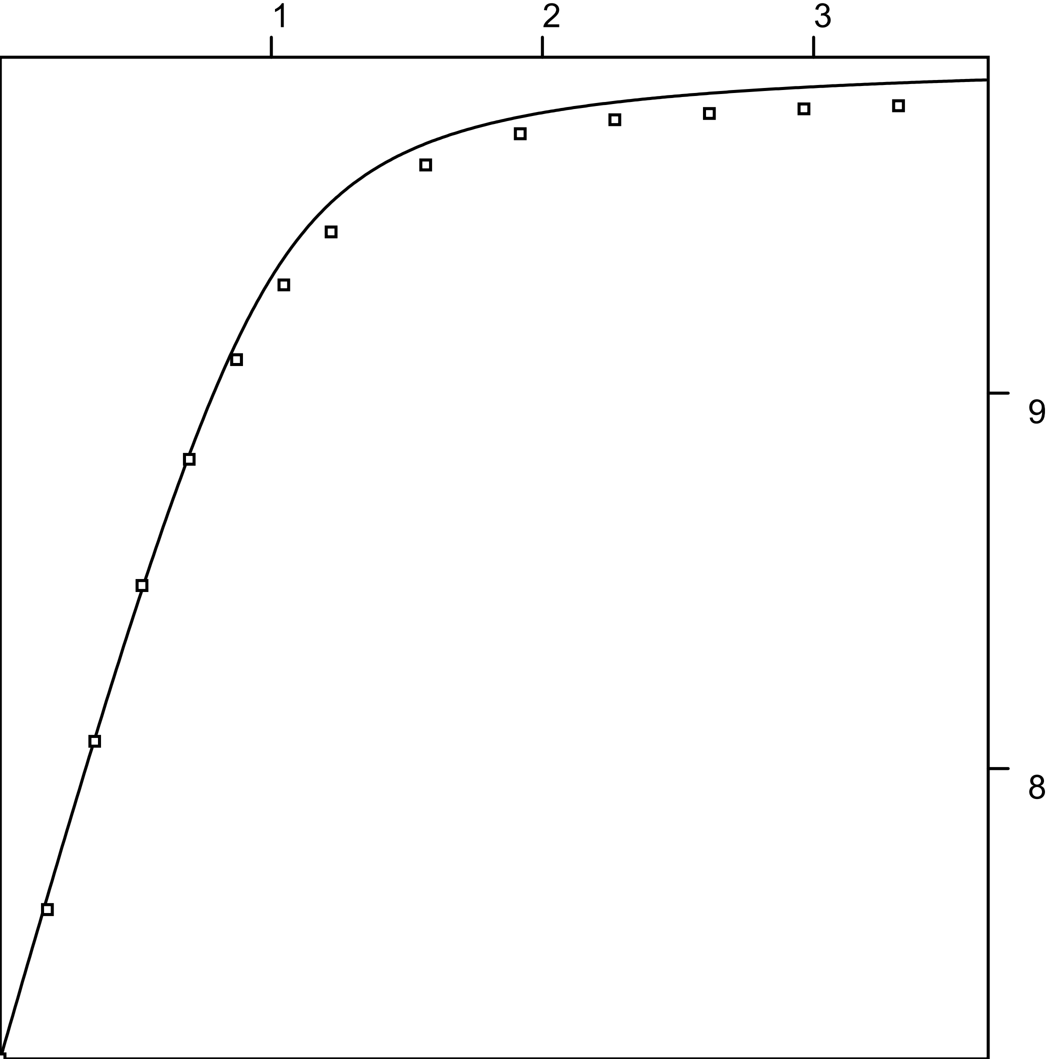

Desplazamiento	Volumen
7.2266	0
7.6243	0.02
8.0725	0.02
8.4874	0.02
8.8235	0.02
9.0891	0.02
9.2882	0.02
9.4293	0.02
9.6077	0.04
9.6907	0.04
9.7281	0.04
9.7446	0.04
9.7571	0.04
9.7654	0.04

Constante de Asociación
Constante: 4.2e+04 1/M
Despl. Max.: 13.491

Host: receptor Id
Peso molecular: 572
Peso puesto (mg): 1.14
Volumen total (ml): 0.5
Volumen puesto (ml): 0.5

Guest: isoftalato
Peso molecular: 648
Peso puesto(mg): 10.3
Volumen puesto(ml): 1

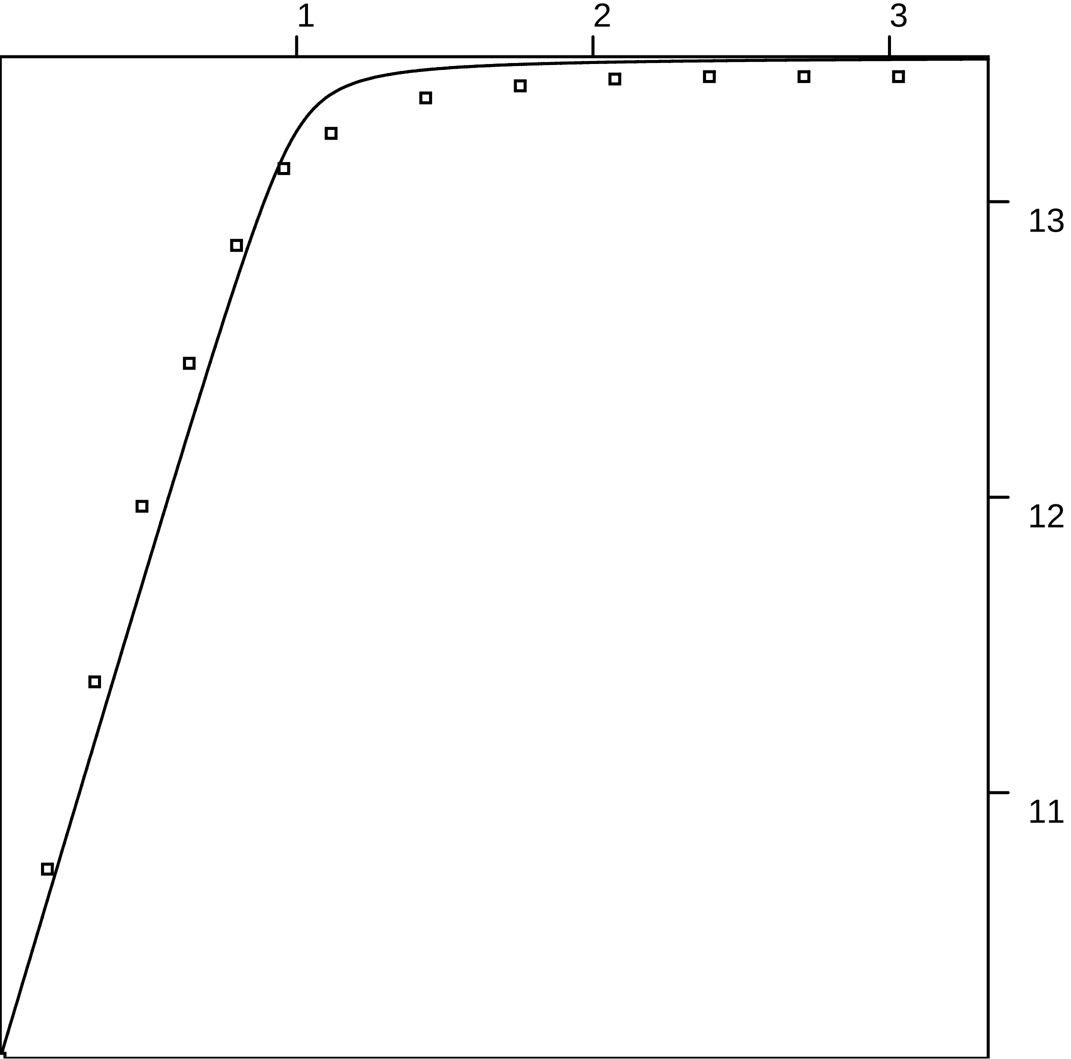

Desplazamiento	Volumen
10.101	0
10.741	0.02
11.375	0.02
11.969	0.02
12.453	0.02
12.852	0.02
13.112	0.02
13.231	0.02
13.351	0.04
13.392	0.04
13.415	0.04
13.423	0.04
13.423	0.04
13.423	0.04

Constante de Asociación
Constante: 1e+04 1/M
Despl. Max.: 13.051

Host: receptor Id
Peso molecular: 572
Peso puesto (mg): 0.75
Volumen total (ml): 0.5
Volumen puesto (ml): 0.5

Guest: tereftalato cloroformo-DMSO
Peso molecular: 648
Peso puesto(mg): 10.6
Volumen puesto(ml): 1

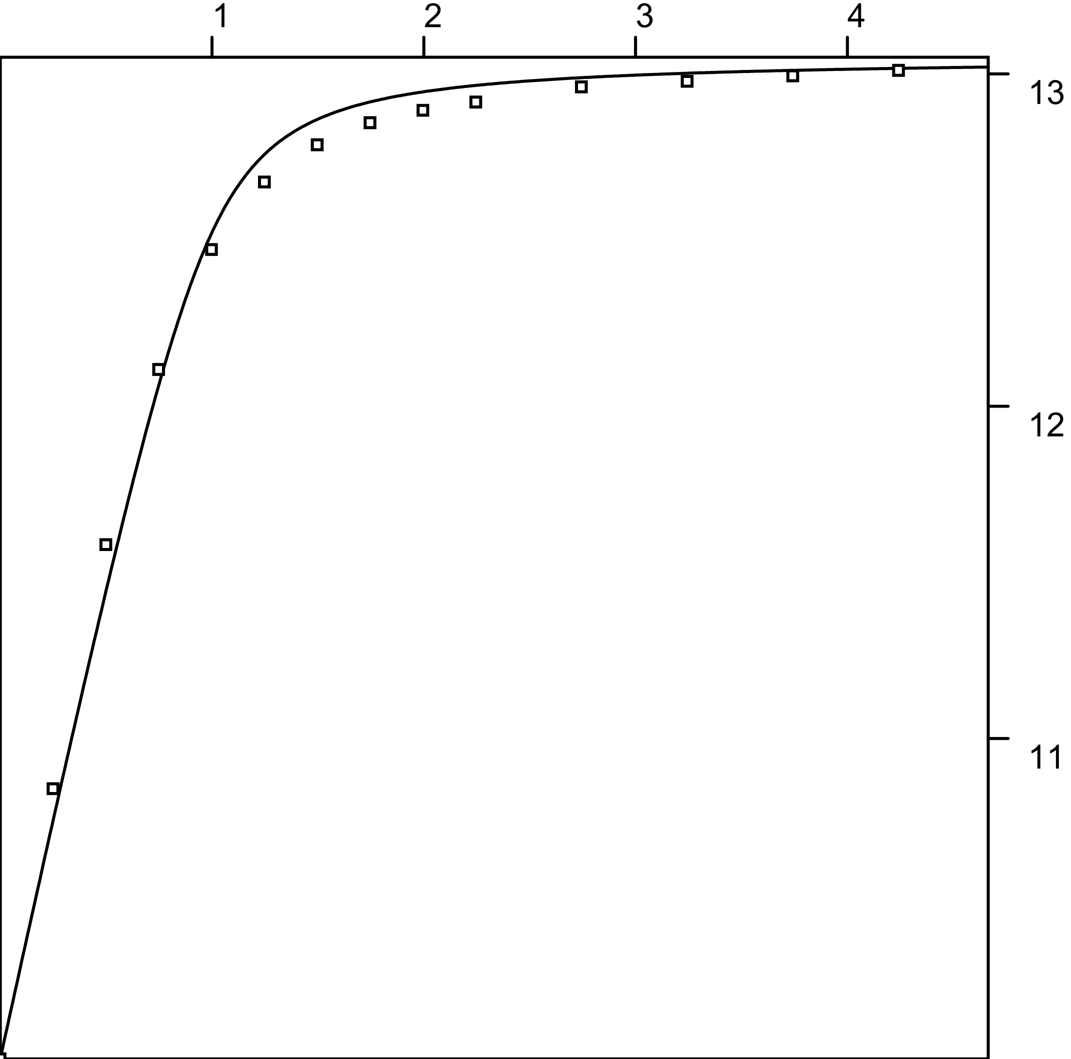

Desplazamiento	Volumen
10.036	0
10.849	0.02
11.583	0.02
12.11	0.02
12.471	0.02
12.675	0.02
12.787	0.02
12.853	0.02
12.89	0.02
12.915	0.02
12.961	0.04
12.977	0.04
12.994	0.04
13.011	0.04

4 b.- Titration curves of **2** with:

4.1. Succinate

4.2. Glutarate

4.3. Adipate

4.4. Pimelate

4.5. Suberate

4.6. Isophthalate

4.7. Terphthalate

Constante de Asociación
Constante: 2.1e+02 1/M
Despl. Max.: 15.57

Host: receptor Ia
Peso molecular: 515
Peso puesto (mg): 1.03
Volumen total (ml): 0.5
Volumen puesto (ml): 0.5

Guest: succinato cloroformo-DMSO
Peso molecular: 600
Peso puesto(mg): 10.6
Volumen puesto(ml): 1

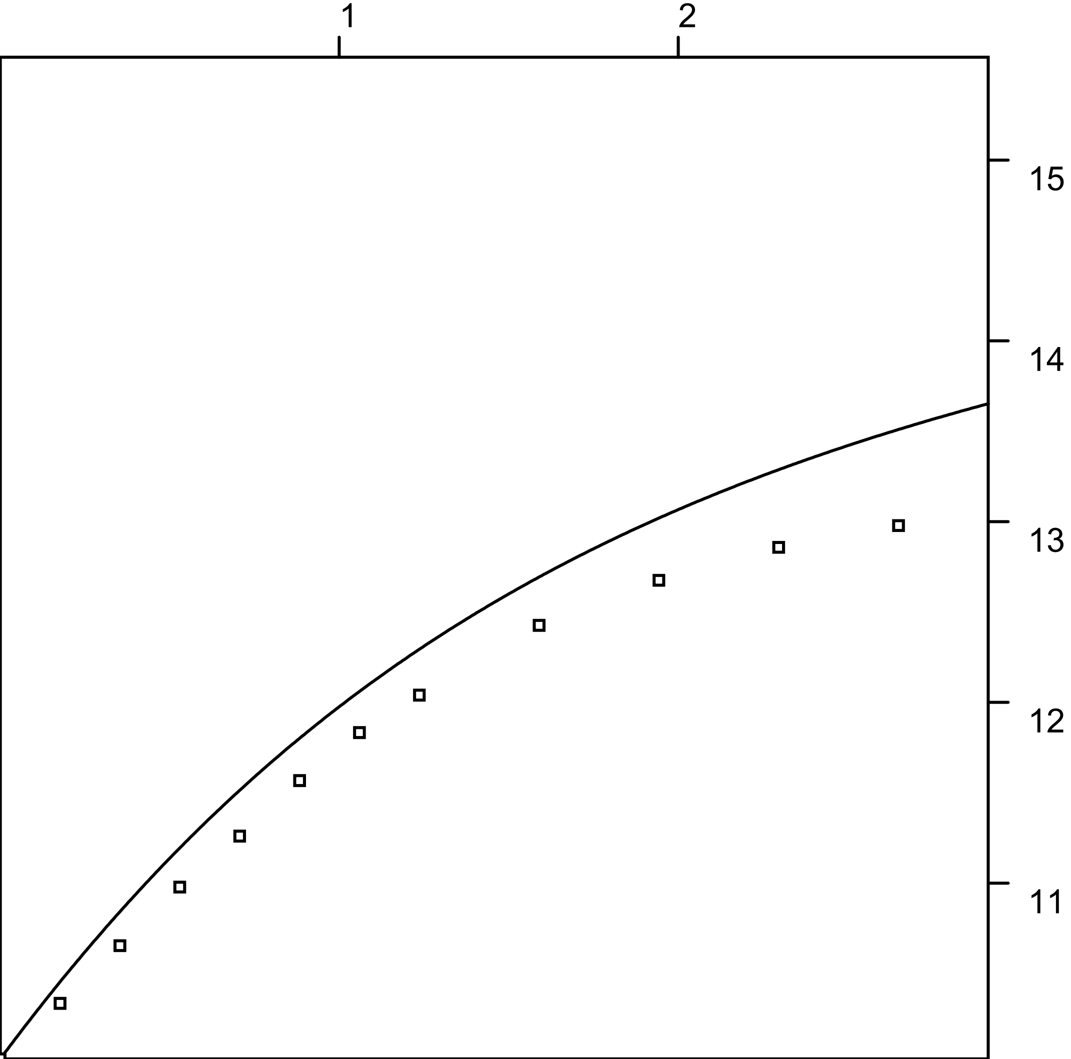

Desplazamiento	Volumen
10.027	0
10.335	0.02
10.654	0.02
10.978	0.02
11.26	0.02
11.567	0.02
11.832	0.02
12.04	0.02
12.426	0.04
12.675	0.04
12.857	0.04
12.978	0.04

Constante de Asociación
Constante: 5.8e+03 1/M
Despl. Max.: 9.763

Host: receptor Ia
Peso molecular: 515
Peso puesto (mg): 1
Volumen total (ml): 0.5
Volumen puesto (ml): 0.5

Guest: glutarato cloroformo- DMSO
Peso molecular: 614
Peso puesto(mg): 10
Volumen puesto(ml): 1

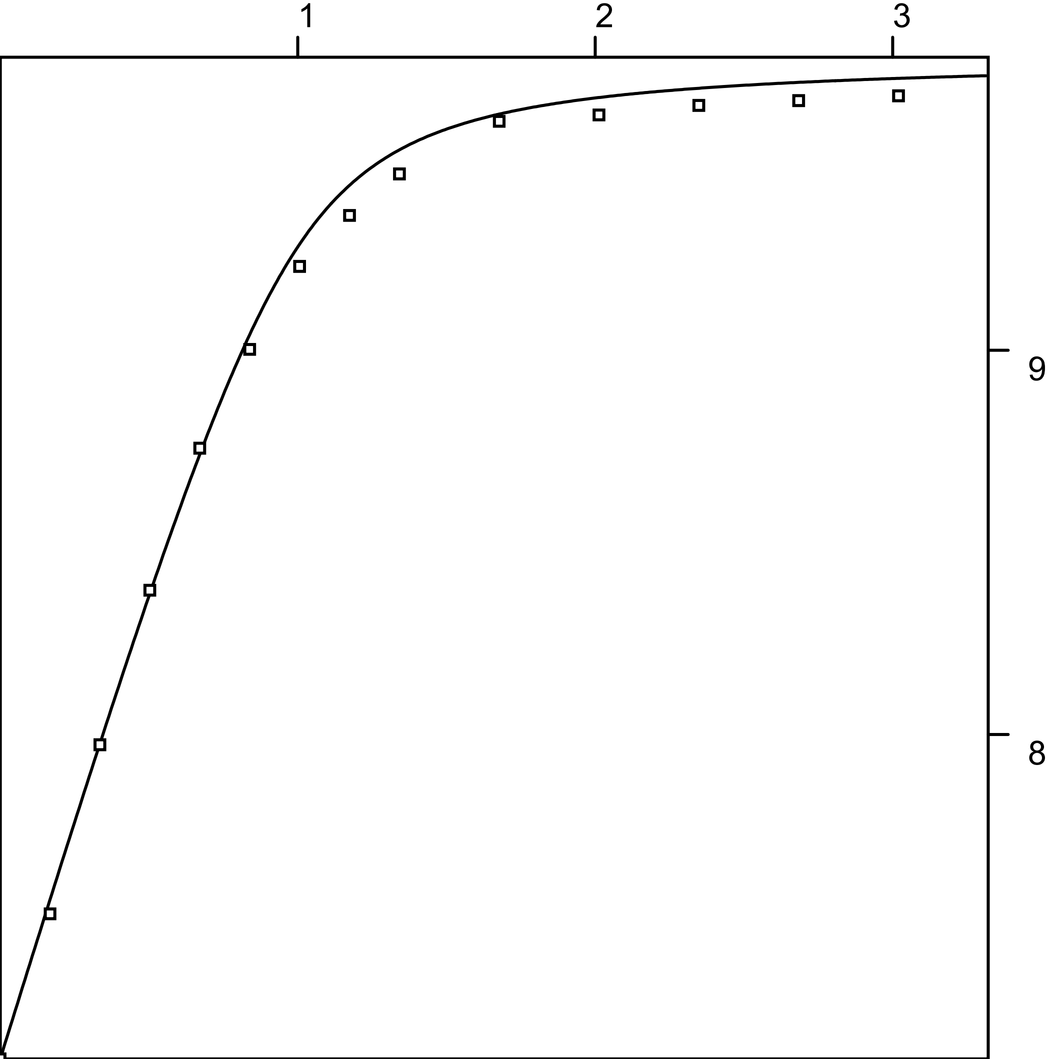

Desplazamiento	Volumen
7.1557	0
7.5333	0.02
7.9731	0.02
8.3753	0.02
8.7449	0.02
9.0021	0.02
9.2179	0.02
9.3507	0.02
9.4586	0.02
9.5955	0.04
9.6121	0.04
9.637	0.04
9.6494	0.04
9.6619	0.04

Constante de Asociación
Constante: 3.9e+03 1/M
Despl. Max.: 9.8127

Host: ia
Peso molecular: 515
Peso puesto (mg): 0.9
Volumen total (ml): 0.5
Volumen puesto (ml): 0.5

Guest: adipato
Peso molecular: 628
Peso puesto(mg): 10.3
Volumen puesto(ml): 1

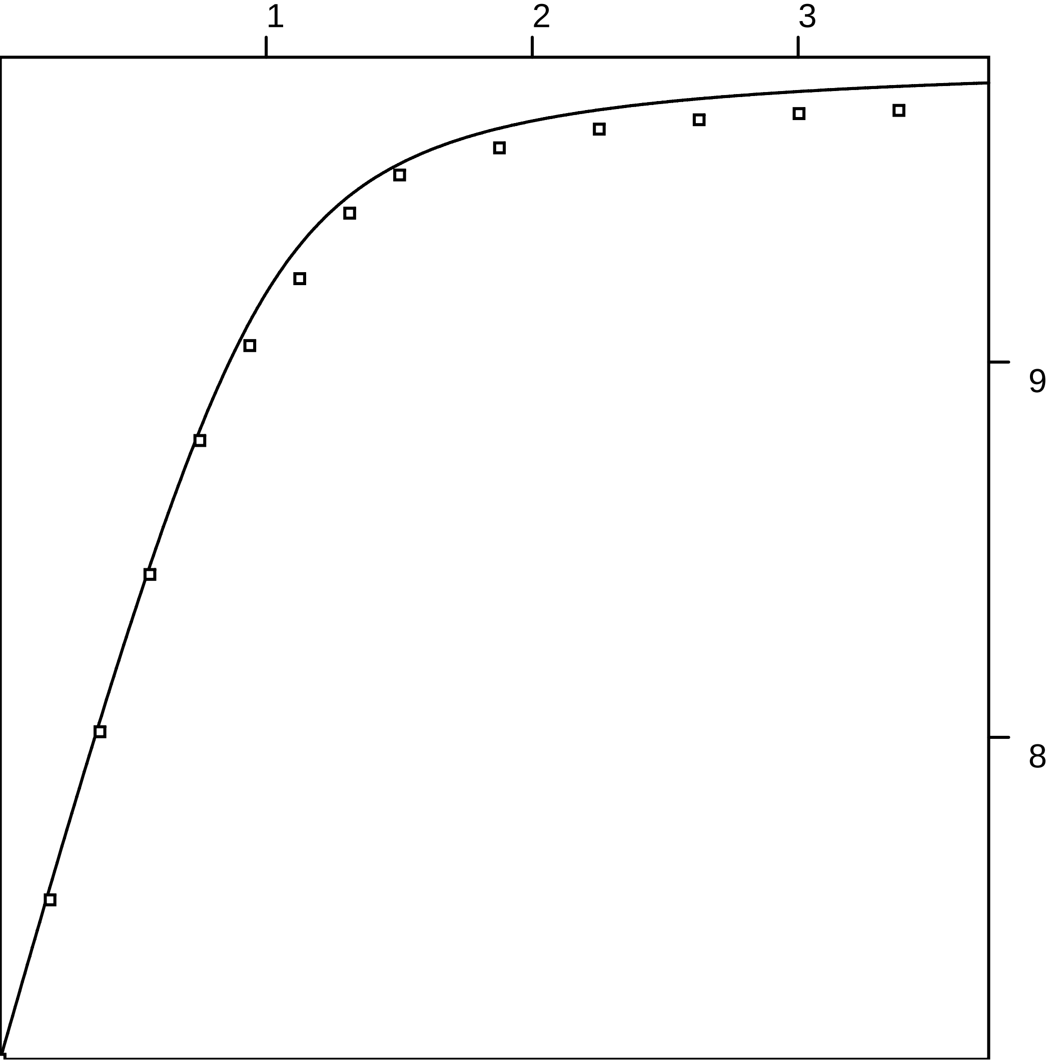

Desplazamiento	Volumen
7.1417	0
7.5665	0.02
8.0146	0.02
8.4337	0.02
8.7905	0.02
9.0436	0.02
9.222	0.02
9.3965	0.02
9.4982	0.02
9.5706	0.04
9.6204	0.04
9.6453	0.04
9.6619	0.04
9.6702	0.04

Constante de Asociación
Constante: 3.1e+03 1/M
Despl. Max.: 9.6474

Host: ia
Peso molecular: 515
Peso puesto (mg): 0.9
Volumen total (ml): 0.5
Volumen puesto (ml): 0.5

Guest: pimelato
Peso molecular: 644
Peso puesto(mg): 9.7
Volumen puesto(ml): 1

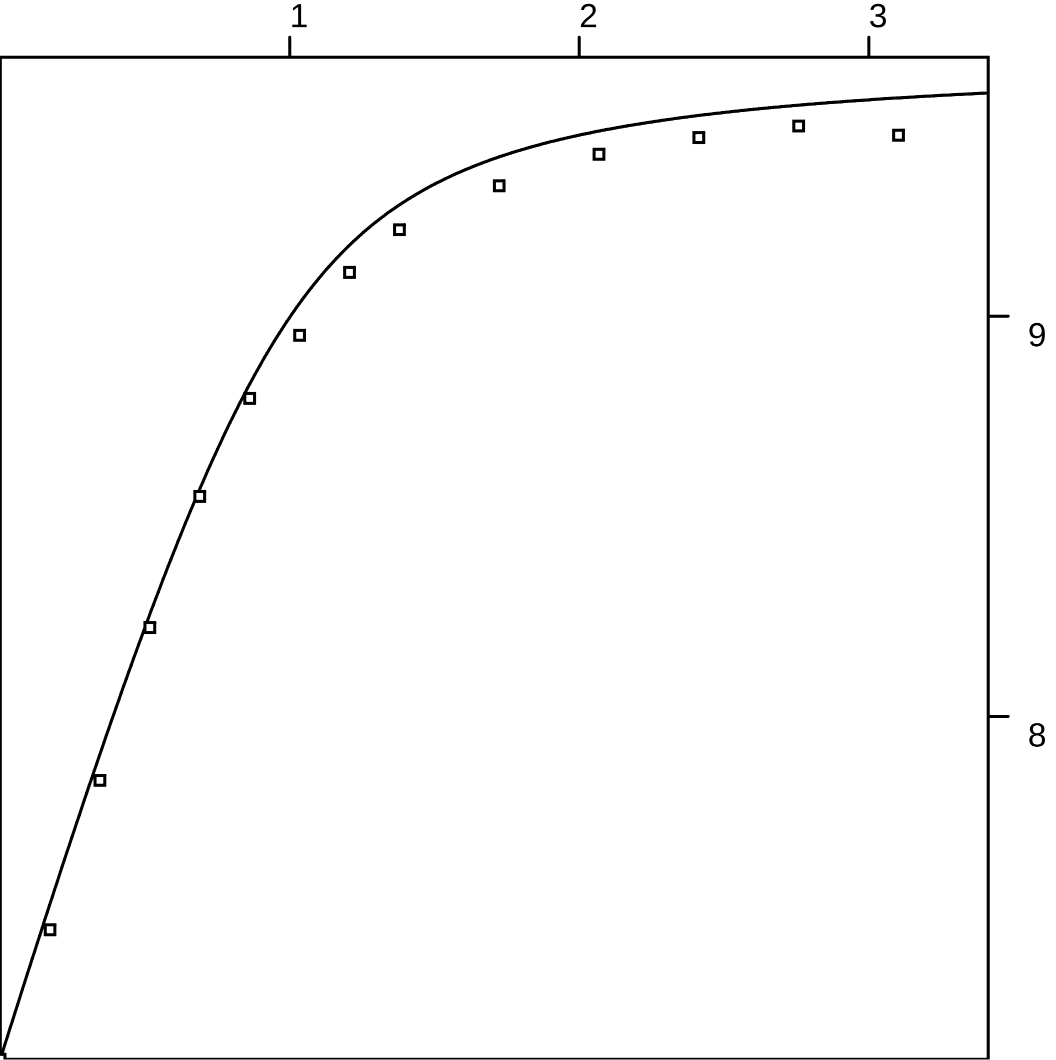

Desplazamiento	Volumen
7.143	0
7.4667	0.02
7.8401	0.02
8.2219	0.02
8.5496	0.02
8.7945	0.02
8.9521	0.02
9.1091	0.02
9.2157	0.02
9.3256	0.04
9.4044	0.04
9.4459	0.04
9.4749	0.04
9.4519	0.04

Constante de Asociación
Constante: 2.8e+03 1/M
Despl. Max.: 13.591

Host: ia
Peso molecular: 515
Peso puesto (mg): 0.99
Volumen total (ml): 0.5
Volumen puesto (ml): 0.5

Guest: suberato
Peso molecular: 656
Peso puesto(mg): 12.5
Volumen puesto(ml): 1

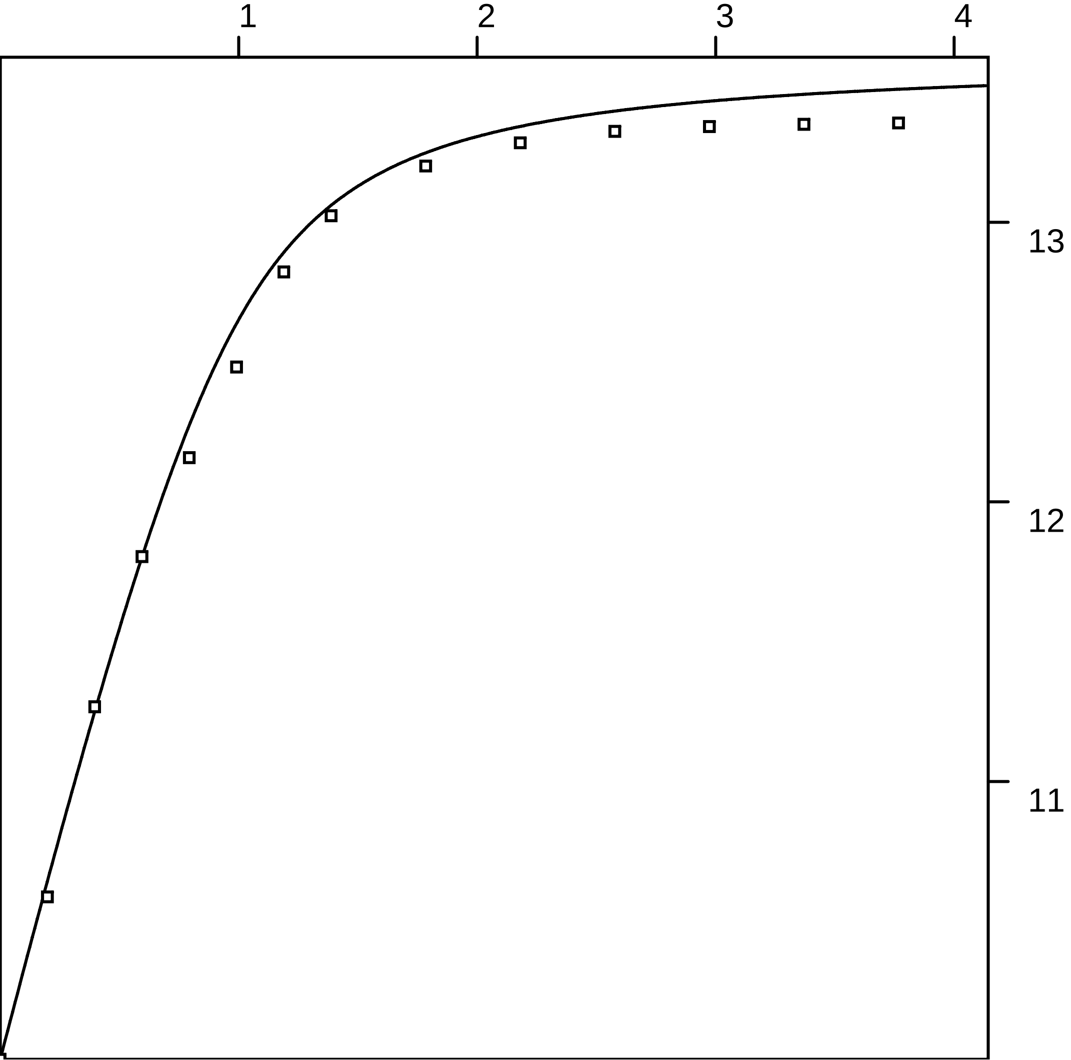

Desplazamiento	Volumen
10.006	0
10.587	0.02
11.267	0.02
11.804	0.02
12.158	0.02
12.482	0.02
12.822	0.02
13.023	0.02
13.201	0.04
13.284	0.04
13.325	0.04
13.342	0.04
13.35	0.04
13.355	0.04

Constante de Asociación
Constante: 1.7e+04 1/M
Despl. Max.: 9.6683

Host: receptor Ia
Peso molecular: 515
Peso puesto (mg): 1.03
Volumen total (ml): 0.5
Volumen puesto (ml): 0.5

Guest: isoftalato
Peso molecular: 648
Peso puesto(mg): 10.3
Volumen puesto(ml): 1

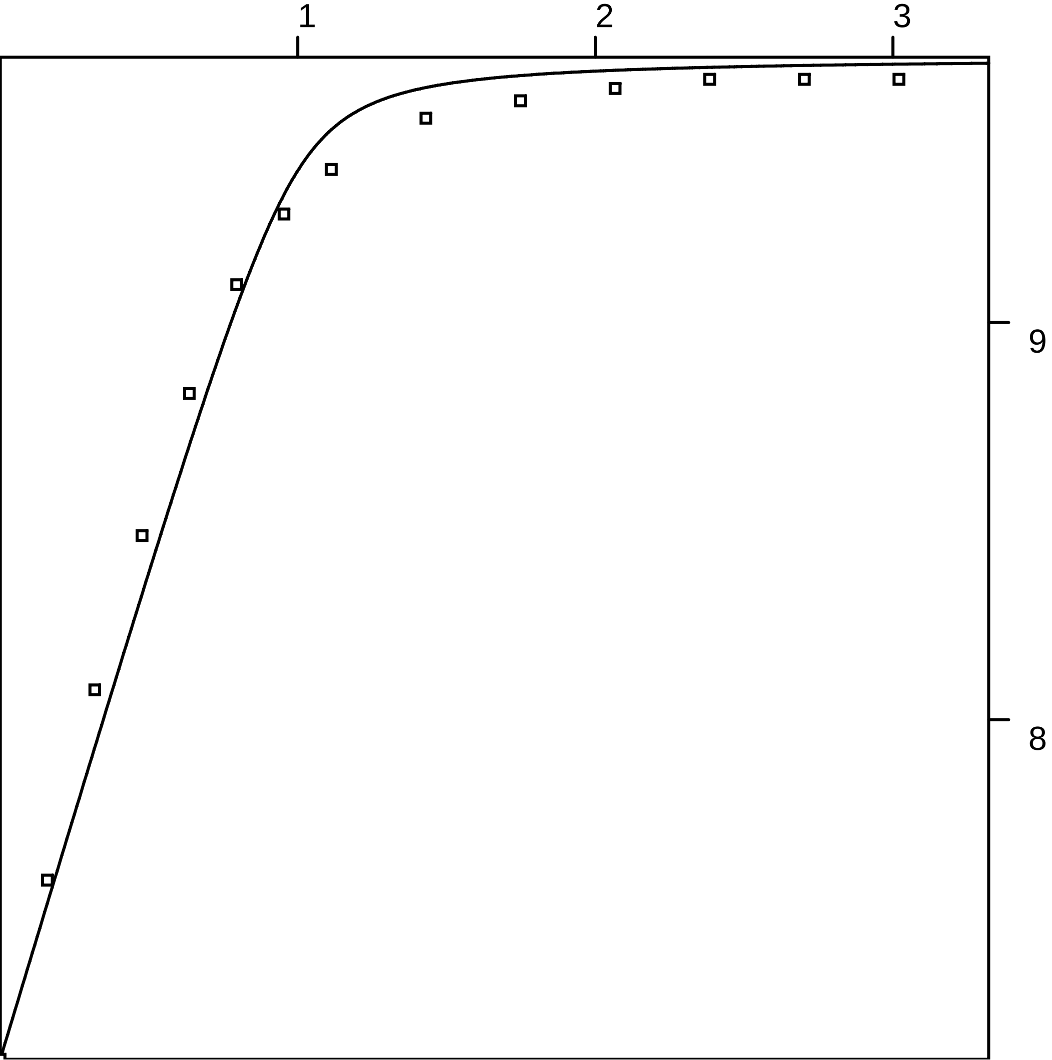

Desplazamiento	Volumen
7.145	0
7.596	0.02
8.075	0.02
8.463	0.02
8.821	0.02
9.095	0.02
9.273	0.02
9.385	0.02
9.514	0.04
9.558	0.04
9.589	0.04
9.612	0.04
9.612	0.04
9.612	0.04

Constante de Asociación
Constante: 6.2e+03 1/M
Despl. Max.: 12.936

Host: receptor Ia
Peso molecular: 515
Peso puesto (mg): 0.85
Volumen total (ml): 0.5
Volumen puesto (ml): 0.5

Guest: tereftalato cloroformo-DMSO
Peso molecular: 648
Peso puesto(mg): 10.6
Volumen puesto(ml): 1

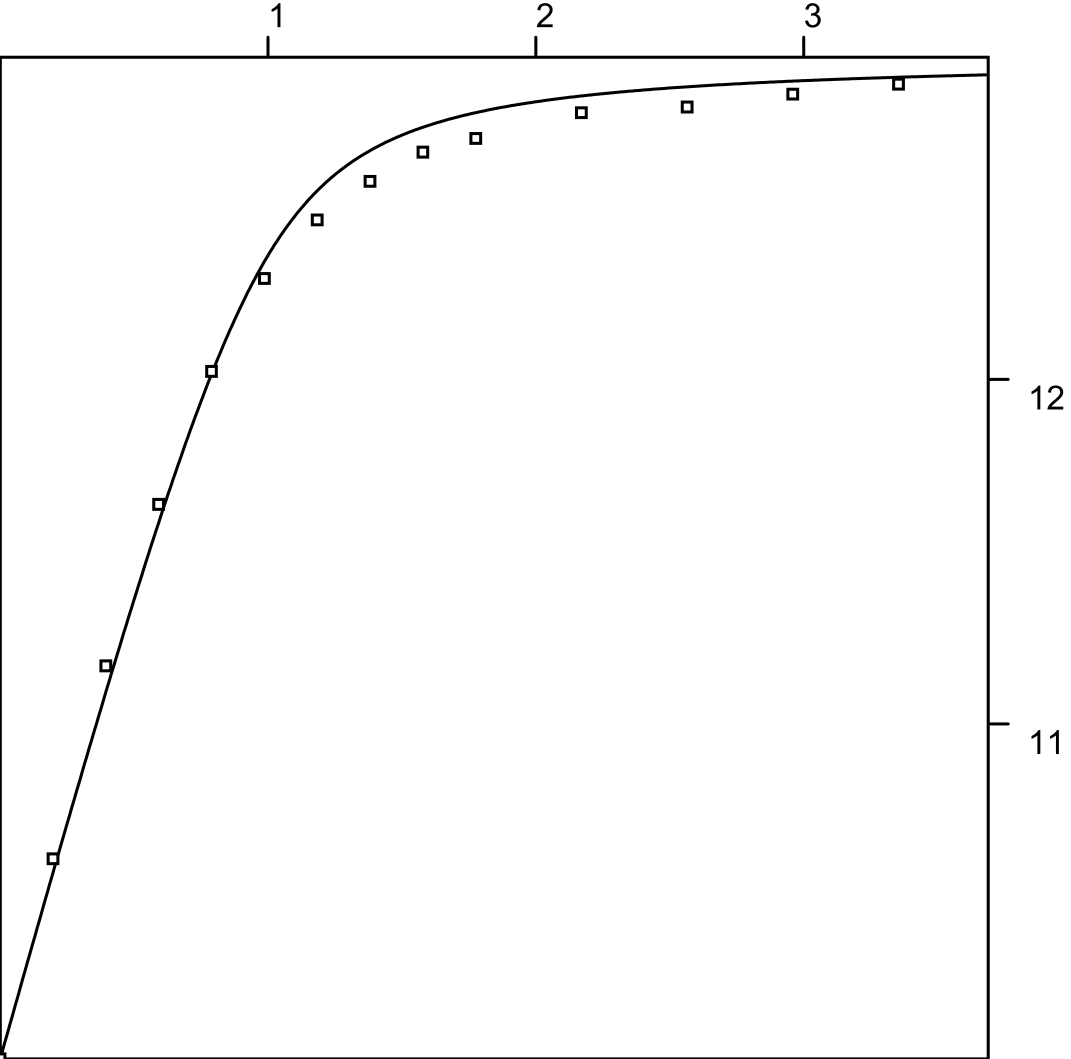

Desplazamiento	Volumen
10.027	0
10.608	0.02
11.168	0.02
11.637	0.02
12.023	0.02
12.293	0.02
12.463	0.02
12.575	0.02
12.66	0.02
12.7	0.02
12.774	0.04
12.791	0.04
12.828	0.04
12.857	0.04

5.- Fluorescence experiments of **1** with glutarate TBA-salt

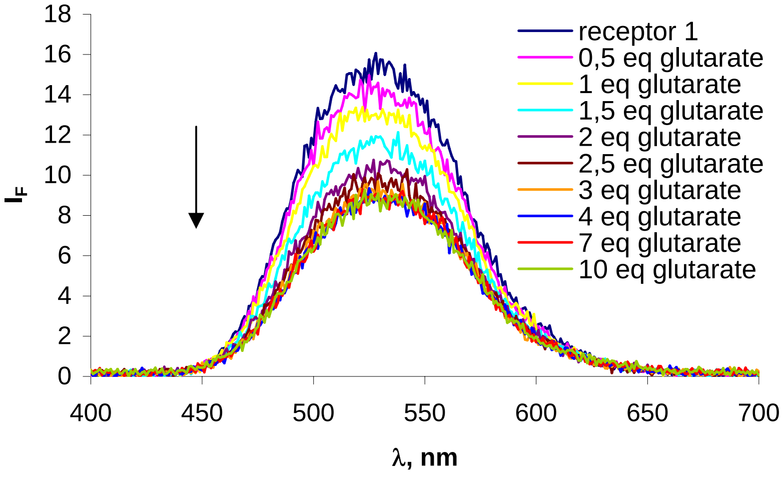

18
16
14
12
10
8
6
4
2
0
I_F
400
450
500
550
600
650
700
λ, nm
receptor 1
0,5 eq glutarate
1 eq glutarate
1,5 eq glutarate
2 eq glutarate
2,5 eq glutarate
3 eq glutarate
4 eq glutarate
7 eq glutarate
10 eq glutarate
